# Genomic characterization of pediatric T-cell acute lymphoblastic leukemia reveals novel recurrent driver mutations

**DOI:** 10.18632/oncotarget.11796

**Published:** 2016-09-01

**Authors:** Jean-François Spinella, Pauline Cassart, Chantal Richer, Virginie Saillour, Manon Ouimet, Sylvie Langlois, Pascal St-Onge, Thomas Sontag, Jasmine Healy, Mark D. Minden, Daniel Sinnett

**Affiliations:** ^1^ CHU Sainte-Justine Research Center, Université de Montréal, Montreal, QC, Canada; ^2^ Princess Margaret Cancer Centre, University Health Network, Toronto, ON, Canada; ^3^ Department of Pediatrics, Faculty of Medicine, Université de Montréal, Montreal, QC, Canada

**Keywords:** T-cell acute lymphoblastic leukemia, X-linked tumor suppressor, MED12, USP9X, U2AF1

## Abstract

T-cell acute lymphoblastic leukemia (T-ALL) is an aggressive hematologic malignancy with variable prognosis. It represents 15% of diagnosed pediatric ALL cases and has a threefold higher incidence among males. Many recurrent alterations have been identified and help define molecular subgroups of T-ALL, however the full range of events involved in driving transformation remain to be defined. Using an integrative approach combining genomic and transcriptomic data, we molecularly characterized 30 pediatric T-ALLs and identified common recurrent T-ALL targets such as *FBXW7, JAK1, JAK3, PHF6, KDM6A* and *NOTCH1* as well as novel candidate T-ALL driver mutations including the p.R35L missense mutation in splicesome factor *U2AF1* found in 3 patients and loss of function mutations in the X-linked tumor suppressor genes *MED12* (frameshit mutation p.V167fs, splice site mutation g.chrX:70339329T>C, missense mutation p.R1989H) and *USP9X* (nonsense mutation p.Q117*). *In vitro* functional studies further supported the putative role of these novel T-ALL genes in driving transformation. *U2AF1* p.R35L was shown to induce aberrant splicing of downstream target genes, and shRNA knockdown of *MED12* and *USP9X* was shown to confer resistance to apoptosis following T-ALL relevant chemotherapy drug treatment in Jurkat leukemia cells. Interestingly, nearly 60% of novel candidate driver events were identified among immature T-ALL cases, highlighting the underlying genomic complexity of pediatric T-ALL, and the need for larger integrative studies to decipher the mechanisms that contribute to its various subtypes and provide opportunities to refine patient stratification and treatment.

## INTRODUCTION

Acute lymphoblastic leukemia (ALL) is the most common childhood cancer, accounting for 25% of all pediatric tumors [1]. Despite continued refinement of childhood ALL subtype classification and improved risk-based treatment strategies, survival rates remain significantly lower among high-risk patients [2]. Pediatric T-cell ALL (T-ALL) represents 10–15% of ALL cases [3] with a quarter of the patients experiencing relapse, and lower post-relapse survival compared to the more common B-lineage ALL [1]. Interestingly, a threefold higher incidence is observed among males [4], however the biological implications underlying this gender bias remain poorly understood. Despite the introduction of intensified chemotherapy protocols, very few inroads into new therapeutic approaches for these high-risk patients have been made. Recent studies [5–8] have shown that further classification of T-ALL could reveal new diagnostic markers and provide alternative targeted treatment options.

Immunophenotypic and gene expression signature analyses revealed a limited number of T-ALL subtypes based largely on differential expression of surface antigen markers and oncogene expression signatures related to stage-specific T-cell developmental arrest [3, 9, 10]. Gene fusions involving the juxtaposition of transcription factor proto-oncogenes under the control of T-cell specific enhancers located in the *TCRB* (7q34) or *TCRA-TCRD* (14q11) have been shown to be essential driver events in T-ALL and further define molecular subtypes [9]. Additional recurrent, as well as cryptic chromosomal rearrangement events that lead to T-cell specific proto-oncogene activation have also been described and some have shown prognostic significance. For instance, CALM-AF10 resulting from the t(10;11)(p13;q14-21) translocation is one of the most frequent fusion events found in 10% of childhood T-ALL cases and has been associated with poor prognosis, particularly among immature T-ALL patients [11, 12].

Recent studies have used comprehensive genomic approaches to gain further insight into the mutational landscape of T-ALL and have led to the identification of novel disease mechanisms [6, 8] and recurrent somatic alterations with pathogenic relevance. The most prevalent are constitutive activation of NOTCH1 signaling, observed in up to 60% of T-ALL patients [13], and loss of the *CDKN2A/p16INK4a* (chromosome 9p21) locus [14], occurring in up to 70% of cases. Loss of function mutations in *FBXW7* are also frequent in T-ALL (about 15% of cases) and contribute to sustained NOTCH1 activation by preventing its proteasomal degradation in the nucleus [15]. Other frequently altered gene/pathway categories in T-ALL include signal transduction (*PTEN, JAK1, JAK3, NF1, NRAS, IL7R* and *FLT3*), transcription factors (*WT1, LEF1, ETV6, GATA3* and *BCL11B*) as well as chromatin remodeling (*EZH2, SUZ12, EED* and *KDM6A/UTX*) [15]. Such studies have also identified a distinct, very aggressive T-ALL subtype defined by very early arrest in T-cell development [16, 17, 5]. These early T-cell precursor ALLs (ETP-ALLs) were genetically characterized by activating mutations in genes regulating cytokine and RAS signaling, inactivating mutations in hematopoietic development genes and histone-modifying genes [5].

These observations suggest that large, integrative efforts will continue to yield further insight into childhood T-ALL, particularly given that the pathogenesis of this disease and of its various subtypes cannot be entirely explained by the data currently available. In this study, we used a combination of exome and transcriptome sequencing, as well as high-density genotyping to characterize 30 childhood T-ALLs. We identified common recurrent mutations in known T-ALL genes (e.g. *NOTCH1, PHF6, FBXW7* and *JAK3*) as well as novel somatic mutations in genes involved in RNA splicing (*U2AF1*), chromatin remodeling (*KMT2C/MLL3*) and of particular interest given the observed male gender bias, in X-linked genes *MED12* and *USP9X*. Additional functional studies provide evidence of the potential implication of these newly identified mutations in childhood T-ALL pathogenesis. Overall, our findings indicate a need for further large-scale genomic investigations to refine patient stratification and optimize treatment strategies in childhood T-ALL.

## RESULTS

### The genomic landscape of childhood T-ALL

**Figure 1:**
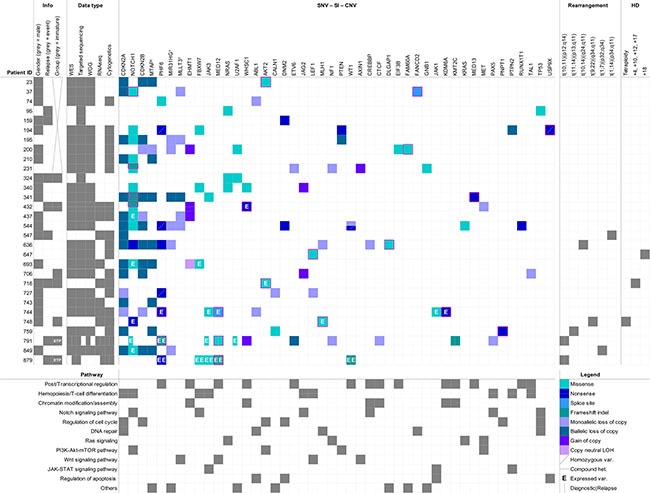
Overview of the SNVs, small indels and structural variations identified among 30 childhood T-ALL patients Genes (top) are ordered according to the number of events identified in the cohort (descending order from left to right). Pathways (bottom) are ordered according to the number of genes identified as mutated (descending order from top to bottom). The “Info” and “Data type” sections respectively inform about the clinical data (gender, relapse status and differentiation group classification) and the type of informative data (WES, targeted sequencing, WGG, RNA-seq, cytogenetics) available for each patient. ETP-ALL cases are indicated directly in the corresponding square of the “Group” column in the “Info” section. SNVs and Indels unreferenced in COSMIC v72 are indicated by a pink frame and were considered as novel. The black cross stands for missing data. †: genes included in the deleted 9p region with tumor suppressor genes *CDKN2A/B*. WES: Whole Exome Sequencing; WGG: Whole Genome Genotyping; SNV: Single Nucleotide Variant; SI: Small Indel; CNV: Copy Number Variant; HD: Hyperdiploidy.

Partially overlapping data from cytogenetic analysis, whole exome and genome sequencing, ultra-deep targeted re-sequencing, and RNA sequencing were available at diagnosis for 30 childhood T-ALL patients (matched normal-tumor) and at relapse for two of these patients (Figure 1 and Table 1). Immunophenotyping and gene expression data, when available, were used to classify patients according to T-cell maturation status (Table 1, Supplementary Figure S1, Supplementary Table S1, S2 Table and Supplementary Information). Seven patients (324, 432, 706, 716, 748, 791 and 879) clustered as early immature T-ALLs, among whom 2 patients (791 and 879) showed immunophenotype and expression markers indicative of an early T-cell precursor ALL (ETP-ALL) phenotype [16]. Thirteen patients (340, 341, 437, 544, 547, 636, 647, 693, 727, 743, 744, 759 and 849) were classified as mature T-ALLs and 10 patients could not be classified due to insufficient data (Table 1).

**Table 1:** Childhood T-ALL patient clinical information

Patient ID	Gender	Cerebrospinal fluid invasion	Blast cells in the blood sample (%)	Blast cells in the BM sample (%)	WBC (.10^9^/l)	Platelet (.10^9^/l)	Treatment protocol	Relapse	Death	Diagnosis age (months)	Time before relapse (months)	Relapse free survival^a^ (months)	T-ALL group
23	M	N	–	85.0^a^	340,3	55,0	DFCI 91–01	N	N	168	NA	244	–
37	M	N	–	92.0^b^	361,0	19,0	DFCI 91–01	N	N	94	NA	225	–
74	M	N	–	60.0^b^	10,9	119,0	DFCI 95–01	N	N	97	NA	79	–
95	F	N	–	–	–	–	FRALLE	Y	Y	66	28, 34	NA	–
159	M	N	–	–	17,5	–	FRALLE	Y	Y	81	10, 13	NA	–
194	M	N	–	100.0^b^	28,8	36,0	DFCI 95–01	N	N	152	NA	213	–
195	F	N	–	90.0^b^	163,0	21,0	DFCI 95–01	N	N	105	NA	29	–
200	M	N	–	71,0	52,1	85,0	DFCI 95–01	N	N	112	NA	89	–
210	M	N	–	93.0^b^	142,0	28,0	DFCI 95–01	N	N	205	NA	39	–
231	F	N	–	92.0^b^	29,3	70,0	DFCI 95–01	N	N	216	NA	214	–
324	M	N	–	–	22,7	136,0	DFCI 95–01	Y	N	86	0, 135	NA	Immature
340	M	N	85,0	94,5	191,5	129,0	DFCI 95–01	N	N	117	NA	193	Mature
341	M	N	33,0	96,5	42,8	62,0	DFCI 95–01	N	N	191	NA	193	Mature
432	F	> 5.10^3^ blasts/ml	89,0	83,0	366,7	285,0	DFCI 2000–01	Y	Y	176	14	NA	Immature
437	M	N	32,0	92,0	181,1	52,0	DFCI 2000–01	N	N	151	NA	125	Mature
544	M	< 5.10^3^ blasts/ml	97,0	99,5	465,6	31,0	DFCI 2000–01	N	N	137	NA	113	Mature
547	M	N	–	90,2	195,0	66,0	DFCI 2000–01	Y	Y	180	9	NA	Mature
636	F	N	88,0	90,5	151,3	70,0	DFCI 2000–01	N	N	179	NA	132	Mature
647	F	N	68,0	92,0	31,8	15,0	DFCI 2000–01	N	N	68	NA	132	Mature
693	M	> 5.10^3^ blasts/ml	66,0	85,5	93,6	99,0	DFCI 2005–01	N	N	162	NA	120	Mature
706	F	< 5.10^3^ blasts/ml	93,0	90,5	253,1	24,0	DFCI 2005–01	N	N	162	NA	101	Immature
716	M	< 5.10^3^ blasts/ml	89,0	95,0	274,4	34,0	DFCI 2005–01	Y	Y	12	19	NA	Immature
727	M	> 5.10^3^ blasts/ml	66,0	90,5	119,4	74,0	DFCI 2005–01	N	N	203	NA	98	Mature
743	M	< 5.10^3^ blasts/ml	89,0	91,5	793,5	18,0	DFCI 2005–01	N	N	152	NA	88	Mature
744	M	< 5.10^3^ blasts/ml	97,0	88,0	95,5	27,0	DFCI 2005–01	N	N	178	NA	76	Mature
748	M	N	19,0	33,4	7,3	100,0	DFCI 2005–01	N	N	192	NA	40	Immature
759	F	N	65,0	70,5	133,0	75,0	DFCI 2005–01	N	Y	192	NA	6	Mature
791	F	N	0/13.0	77.7/85.0	27.7/4.28	112.0/102.0	DFCI 2005–01	Y	N	195	57	NA	ETP-ALL
849	M	N	61,0	86,0	105,2	31,0	DFCI 2005–01	N	N	68	NA	46	Mature
879	F	< 5.10^3^ blasts/ml	41.0/18.0	83.0/55.0	3.1/3.9	38.0/49.0	DFCI 2011	Y	N	137	32	NA	ETP-ALL

a Period during which the patient was followed after diagnosis and presented no relapse event.

b Blast counts estimated from genotyping data analysis (Methods). BM: bone marrow; WBC: white blood cell; NA: not applicable; N: no; Y: yes; ETP: early T-cell precursor ALL; (–): missing data.

We identified several structural chromosomal abnormalities within our cohort (Figure 1 and Supplemental Information). Mature T-ALL patients 547, 759 and 636 were shown to carry translocations t(1;14)(p34;q11), t(11;14)(p13;q11) and t(10;14)(q24;q11) respectively, leading to the juxtaposition of the *TAL1*, *LMO2* and *TLX1/HOX11* oncogenes to the T-cell receptor alpha/delta (*TCRA/D*) at locus 14q11 [18–20]. We also identified a rarer translocation t(1;7)(p32;q34)/TRB-TAL1 in the mature T-ALL patient 849 [9] as well as the well-known t(10;11)(p12;q14) CALM-AF10 translocation in both ETP-ALL cases 791 and 879 and a t(9;22)(q34;q11.2)/BCR-ABL in the immature T-ALL patient 748 which is very rare in T-ALL (∼1%) [21, 3]. Above 80% of the patients with available expression data showed activation of at least one (proto-)oncogene such as *TAL1, TLX3, FLT3, LMO2, LYL1* and *PIM1* with no evidence of a related fusion event (Supplementary Figure S1A and Supplementary Table S2). For example, *LMO2* was shown to be upregulated in the mature T-ALL patient 547 and four early immature T-ALL patients including both ETP-ALLs (432, 748, 791 and 879), while none of these patients were identified as carriers of a *LMO2* activating translocation. *LYL1* was upregulated in all but one early immature T-ALL patient (716) without an associated translocation.

On average, we identified 29 somatic SNVs/indels and 37 somatic CNVs per tumor (Figure 1 and Supplementary Table S3). Based on strict filtering criteria (Methods), we identified a total of 68 candidate driver SNVs/indels (55 distinct mutations) across 28 genes among the 30 pediatric T-ALL patients and all patients harboured at least one candidate driver mutation (Figure 1 and Supplementary Table S3). RNA-seq data, when available, confirmed expression of 84% of the mutated alleles (21/25) (Figure 1, Supplementary Figure S2). “Hemopoiesis/T-cell differentiation” was the most frequently altered pathway among the cohort with 80% of patients carrying mutations in 9 genes affecting this pathway. “Post/Transcriptional regulation” (14 genes), “Chromatin modification/assembly” (6 genes), “Notch signaling” (6 genes) and “Regulation of cell cycle” (6 genes) were also found to be frequently altered. 34 of the reported candidate driver mutations were previously reported (COSMIC 72) among which 29 in hematopoietic malignancies (COSMIC v72) including 26 in known T-ALL driver genes such as *FBXW7, JAK1*, *JAK3*, *PHF6, KDM6A* and *NOTCH1.* These variations were mostly clonal (mean variant allele frequency-VAF = 0.48, standard deviation-SD = 0.10), confirming their presence in the majority of tumor cells at diagnosis and their initiating role in T-ALL (Supplementary Figure S3 and Supplemental Information). Ras pathway mutations had significantly lower frequencies compared to these common drivers with a mean VAF = 0.33 (SD = 0.11) (*p* = 0.006, Mann-Whitney-*U* test) (Supplementary Figure S3 and Supplementary Information). Subclonality of these mutations corroborates previous reports [22–24] that describe a secondary role for Ras mutations in T-ALL occurring later in tumor progression. Fifteen patients (50%) harbored *NOTCH1* mutations and two thirds (12/18) of identified *NOTCH1* mutations were located in exons 26 and 27, coding for the extracellular heterodimerization domain and were mainly missense mutations (11/12). The remaining 6 *NOTCH1* mutations were located in exon 34 coding for the C-terminal PEST (4/6) and transactivation (2/6) domains and consisted mainly of truncating mutations (5/6), as previously reported [25]. Recurrent events also involved the tumor suppressor locus 9p21 with *CDKN2A/2B* (*p16INK4a* and *p15INK4b*) deletions occurring in 17 (57%) of our childhood T-ALL cases (Figure 1 and Supplementary Table S3), of which 14 were biallelic. For 5/17 patients, the 9p21 deletion event included the hematopoiesis regulator MLLT3 [26], 6/17 included the long non-coding RNA MIR31HG recently shown to regulate *CDKN2A* expression [27], and 9/17 included the methylthioadenosine phosphorylase MTAP. Although the associations require validations in larger cohorts, patients mutated for *CDKN2A* had significantly less chance of relapse (*p* = 0.0121, Fisher's Exact test). Interestingly, immature T-ALL patients (early immature T-ALL and ETP-ALL together) had significantly less *CDKN2A* alterations (*p* = 0.0044, Fisher's Exact test) and a higher risk of relapse (*p* = 0.0072, Fisher's Exact test).

Twenty-one variations had not previously been reported and thus were considered as novel T-ALL driver mutations, including 3 in *NOTCH1* (p.N1603K, p.P2475fs and p.L2326fs) as well as novel predicted deleterious mutations in the well-known X-linked *PHF6* (p.E221* and p.G226fs) and *KDM6A/UTX* (p.Q692*) [8, 28] (Figure 1 and Supplementary Table S3). Among these novel putative childhood T-ALL drivers, we identified a number of mutations in chromatin remodeling genes *EHMT1* (*n* = 4), *WHSC1* (*n* = 3) and *KMT2C/MLL3* (*n* = 1), a recurrent missense mutation (p.R35L) in the first zinc finger of splicing factor *U2AF1* (*n* = 3), as well as loss of *ABL1* (*n* = 2) and in *MLH1* (*n* = 2), both involved in DNA repair. Two patients had gain of copy of *JAG2* which functions in the Notch signaling pathway, and two novel missense mutations (p.Q79K and p.R208K) were identified in the oncogenic serine/threonine-protein kinase AKT2 involved in cell cycle regulation. We also identified two novel candidate driver genes on the X chromosome: a novel nonsense mutation in the deubiquitinating protease USP9X (p.Q117*) in male patient 194; and 3 novel mutations in MED12, a member of the Mediator complex involved in regulating RNA polymerase II-dependent transcription, were found in male patient 744 (splice site mutation g.chrX:70339329T>C) and female ETP-ALL patients 791 (missense mutation p.R1989H) and 879 (frameshift insertion p.V167fs). Mutations in *USP9X* and *MED12* presented VAFs (mean = 0.97, SD = 0.04) that were similar to the known driver mutations in *PHF6* and *KDM6A/UTX* (mean = 0.97, SD = 0.05) (*p* = 1.0000) (Supplementary Figure S3 and Supplementary Information).

### The novel U2AF1 p.R35L mutation alters pre-mRNA splicing in human T-cells

**Figure 2:**
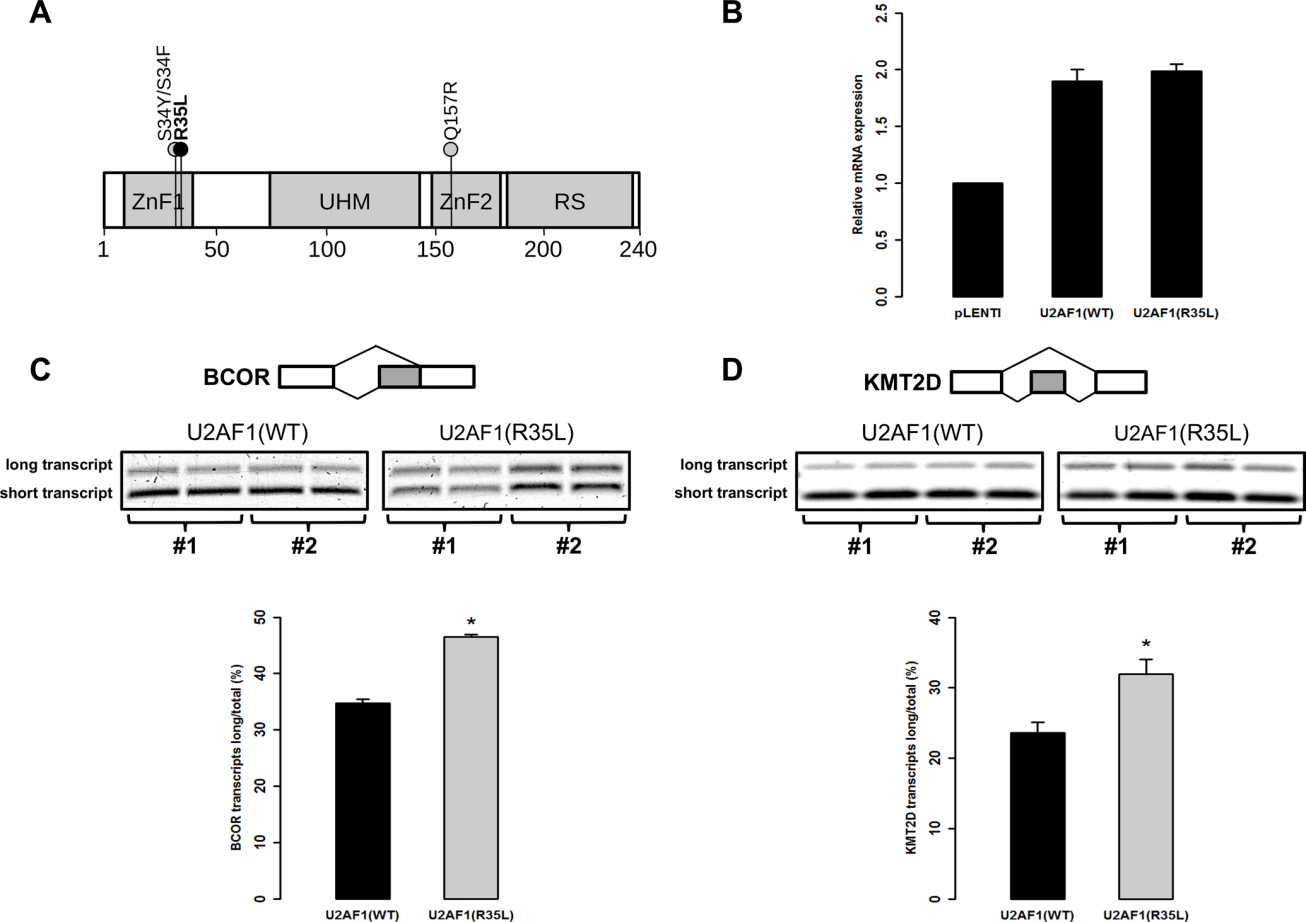
Mutation p.R35L in *U2AF1* alters pre-mRNA splicing in human T-cells (**A**) Schematic representation of U2AF1 predicted protein including zinc fingers 1 and 2 (ZnF1, ZnF2), the U2AF homology motif (UHM) and the arginine-serine rich domain (RS). The black circle indicates the location of the new mutation p.R35L identified here in 3 T-ALL cases. The grey circles indicate previously identified recurrent mutations in U2AF1. The amino acid scale is indicated at the bottom. (**B**) The expression of WT or R35L *U2AF1* transgenes in Jurkat cells was measured by qPCR and reported to the expression of the endogeneous gene measured in cells infected with the empty vector (pLENTI). The alternative splice site utilization of BCOR (**C**) and KMT2D/MLL2 (**D**) mRNAs were measured in infected cells as previously described [32]. RT-PCRs were performed in duplicate on cDNAs obtained from two different infections (#1 and #2) of WT or R35L *U2AF1* transgenes in Jurkat cells and PCR products were electrophoresed on agarose gel stained with SYBR Safe (top left). Quantification of each isoform was done by densitometry using Image J software (version 1.49) and the mean ratio of the long isoforms of BCOR and KMT2D/MLL2 reported to the total of both long and short isoforms (right). Statistical significance was determined by a two-tailed Mann-Whitney *U* test; *P*-values < 0.05 are represented by one asterisk.

The novel recurrent p.R35L missense mutation was located in a zinc finger (ZnF) domain of the *U2AF1* (U2 small nuclear RNA auxiliary factor 1) gene, coding for a member of the spliceosome machinery involved in pre-mRNA processing (Figure 2A and Supplementary Table S3). Three patients (200, 324 and 791) carried the predicted damaging mutation; patient 200 was unclassified and the other two were classified as early immature T-ALLs (324 and the ETP-ALL 791) and experienced relapse. An additional cohort consisting of 8 adult relapsed T-ALL patients was used for further screening of newly identified somatic driver candidates and revealed a clonal p.R35L mutation (VAF = 0.53) in the relapse genome of a 32 years old female case (patient P6, Methods and Supplementary Table S4).

The previously identified *U2AF1* p.S34F mutation (Figure 2A), located in the same ZnF in myelodysplastic syndrome (MDS) patients, was shown to disrupt splicing of a number of cancer-relevant genes leading to overall dysregulation of several downstream pathways including epigenetic regulation and DNA damage response [29, 30–32]. To assess the putative impact of the novel p.R35L mutant on alternative splice site utilization, we tested for the presence of quantifiable isoforms among known U2AF1 targets (*BCOR*, *KMT2D/MLL2*, *KDM6A/UTX* and *PICALM*) [32] in human T lymphocyte (Jurkat) cells. *BCOR* and *KMT2D/MLL2* were the only target genes with two identifiable isoforms (data not shown). Using site-directed mutagenesis, we created mutant T-cell lines overexpressing either WT or p.R35L mutated *U2AF1* (Figure 2B) and showed alternative splice site usage at both *BCOR* and *KMT2D/MLL2* loci that were specific to p.R35L (*p* = 0.0286 for both genes, Mann-Whitney *U* test) (Figure 2C). These data suggest a comparable effect for the p.R35L mutation leading to disrupted splicing and further support a role for functional *U2AF1* mutations in abnormal hematopoiesis, including childhood T-ALL.

### Novel X-linked drivers of T-ALL

**Figure 3:**
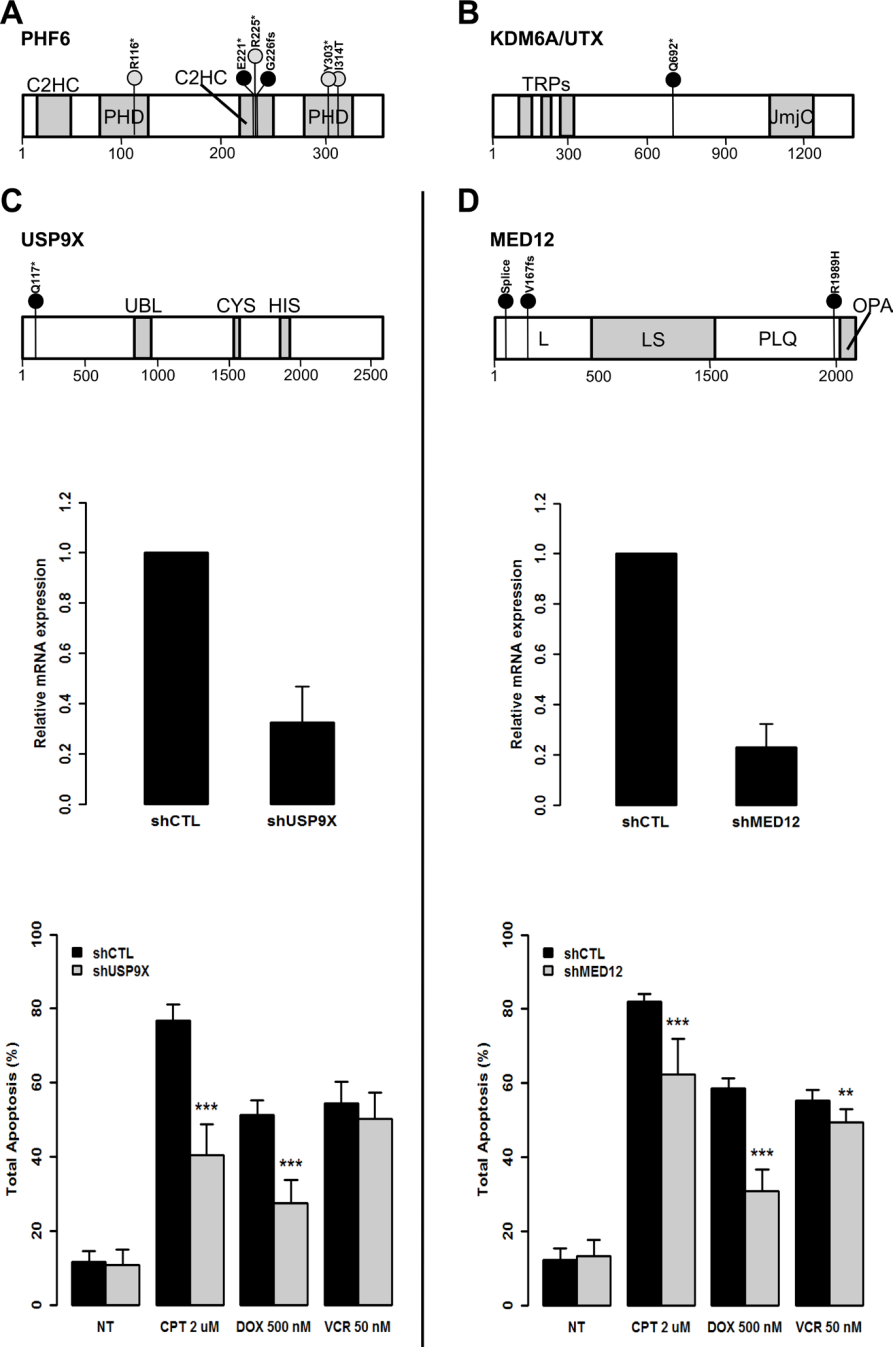
The loss of function of USP9X and MED12 protects from induced apoptosis in leukemic T-cells (**A**) Schematic representation of PHF6 predicted protein including the two PHD type zinc finger domains (PHD) and the two CysCysHisCys type zinc finger domains (C2HC). Black circles indicate the location of new mutations. Grey circles indicate previously referenced mutations identified in this childhood T-ALL cohort. The size of the bars are representative of mutation frequencies in the cohort (*PHF6* p.R225* was identified in three cases while all others were identified in one case). The amino acid scale is indicated at the bottom. (**B**) Schematic representation of KDM6A/UTX predicted protein including three tetratricopeptide repeat domains (TRPs) and the Jumanji C domain (JmjC). (**C**, top) Schematic representation of USP9X protein including the ubiquitin-like module (Ubl) and the USP-definitive cysteine and histidine box catalytic motifs (CYS and HIS). (**D**, top) Schematic representation of MED12 including the leucine-rich domain (L), the leucine- and serine-rich domain (LS), the proline-, glutamine-, leucine-rich domain (PQL) and the opposite paired domain (OPA). ShRNAs were used to knockdown expression of: *USP9X* (**C**, middle) and *MED12* (**D**, middle) in Jurkat cells. Residual mRNA levels after shRNA transduction were measured by RT-qPCR. GAPDH was used as calibrator and non-mammalian shRNAs (shCTL) were used as control for normalization. *In vitro* apoptosis assays show overall reduced levels of apoptosis associated with knockdown of *USP9X* (**C**, bottom) and *MED12* (**D**, bottom). DNA damage-induced apoptosis was provoked with 2 μM of Camptothecin (CPT) for 17 hours or 500 nM of Doxorubicine (DOX) for 18 hours or 50 nM of Vincristine (VCR) for 24 hours. Non-treated (NT) transduced cells were used as controls. Overall apoptosis levels were measured after 30 minutes of annexin V (AnV) propidium iodide (PI) double staining (AnV+/PI–, AnV+/PI+, and AnV–/PI+). Statistical significance was determined by a two-tailed Mann-Whitney *U* test; **P-values < 0.01; ***P-values < 0.001.

In 8 T-ALL patients (74, 194, 544, 636, 727, 744, 791 and 879), we identified 4 known somatic mutations (p.I314T, p.R116*, p.R225*, p.Y303*) and 2 novel mutations (p.E221*, p.G226fs) in the well-known X-linked driver gene *PHF6* (Figure 3A). One mature T-ALL (744) also carried a novel nonsense mutation in *KDM6A/UTX* (p.Q692*) which was recently characterized as a T-ALL X-linked driver gene [8, 28] (Figure 3B). We observed no gender bias in the distribution of *PHF6* mutations in our cohort (*p* = 1.0000) with 25% (5/20) of male patients harboring mutations compared to 30% (3/10) of females. This is in line with recent observations [25, 33] that *PHF6* alone is unlikely to account for the higher incidence of T-ALL among males.

Our genomic investigation revealed 2 new candidate X-linked drivers of T-ALL: *USP9X* and *MED12*. For *USP9X*, we identified a novel truncating mutation (p.Q117*) in exon 5 carried by the unclassified male patient 194 suggesting a tumor suppressor role (Figure 3C).

**Figure 4:**
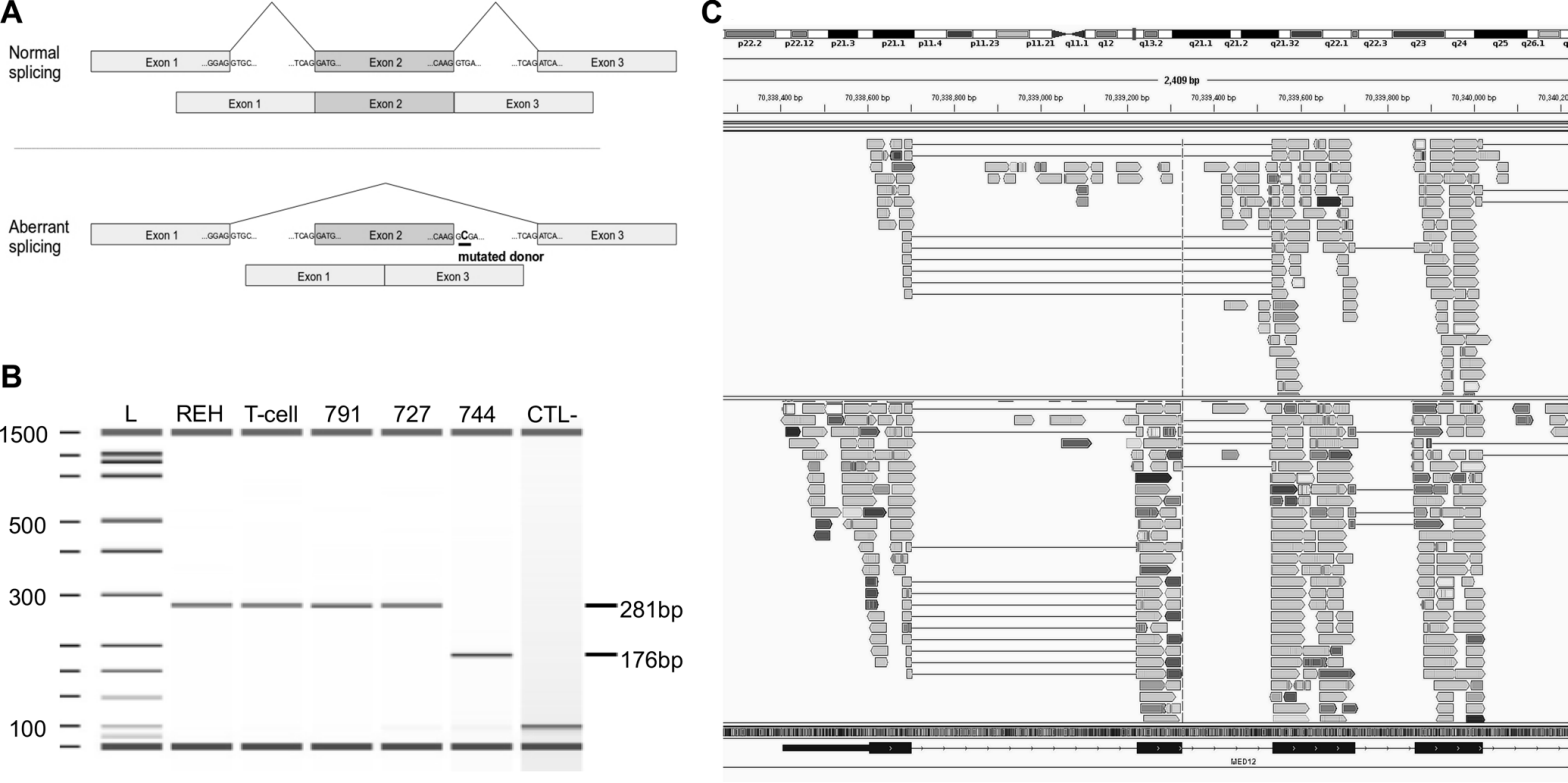
Mutation g.chrX:70339329T>C in *MED12* causes the splicing of exon 2 (**A**) Schematic representation of the normal (top) and aberrant splicing (bottom) of MED12 transcripts from the normal and the identified mutant gene. In the presence of the mutation, the donor splice site of exon 2 (underlined) is skipped and exon 1 is directly spliced to exon 3, leading to an aberrant mature form of MED12 mRNA lacking exon 2. (**B**) Synthetic electrophoresis gel of MED12 PCR products (Methods). cDNAs were synthesized from mature RNA extracted from patient 744 mutated at g.chrX:70339329T>C, REH cells, mature T-cells (CD19-CD3+) isolated from cord blood samples and two T-ALL patients, WT for this position (791 and 727). Amplified fragments of 281 bp (WT samples) and 176 bp (mutated sample) were analyzed using Agilent 2100 Bioanalyzer. The 105 bp difference corresponds to the skipped exon 2. L: ladder; CTL-: negative control. (**C**) Screenshot of the Integrative Genomics Viewer (IGV) window presenting aligned RNA-seq data obtained from cases 744 (top) and a third T-ALL WT patient (693, bottom). The dotted line is centered on the donor splice site of exon 2 of *MED12* (g.chrX:70339329). Case 744 shows aberrant splicing of exon 2, while 693 was WT for this position and shows a mature transcript that includes the exon 2.

As for *MED12*, we identified 3 novel somatic mutations (Figure 3D). One was located at the donor splice site of exon 2 (g.chrX:70339329T>C) in mature male patient 744 (Figure 4A). RNA-seq performed at diagnosis, as well as RT-PCR assays, confirmed that the mutated donor site was skipped leading to an aberrant form of the mature MED12 mRNA where exon 2 was lost (Figure 4B and 4C). This is the first *MED12* mutation shown to lead to exon skipping in cancer. Interestingly, we also identified a missense mutation (p.R1989H) in exon 41 and a frameshift mutation (p.V167fs) in exon 14 of *MED12* in the two female ETP-ALL cases 791 and 879, respectively. While, *MED12* was previously shown to be subject to X-inactivation, it has also been shown that inactivation can be tissue-specific [34]. Through allelic expression analysis, we showed biallelic expression of a germline synonymous SNP (rs5030619) in the female case 879 (Supplementary Figure S4), suggesting that *MED12* could indeed escape X-inactivation in T-ALL.

To further test the impact of the loss of function of *MED12* and *USP9X* in driving T-ALL development or maintenance, we performed *in vitro* small hairpin RNA (shRNA) assays in human T lymphocyte (Jurkat) cells (Figure 3C and 3D) and assessed aberrant proliferation and apoptosis resistance. While no significant changes in proliferation were observed (Supplementary Figure S5), knockdown of both genes led to a reproducible and significant reduction of apoptosis following Camptothecin (CPT) and at least one relevant chemotherapy drug treatment, Doxorubicine (DOX) or Vincristine (VCR), compared to control (Figure 3C and 3D and Supplementary Figure S6) (*p* = 3.375E-06 and 4.114E-05 after CPT, *p* = 4.095E-04 and 4.114e-05 after DOX, *p* = 3.767E-01 and 5.636E-03 after VCR for shUSP9X and shMED12 cells respectively, two-tailed Mann-Whitney *U* test). Although the observed effect might be context-dependent or cell-type specific, these results provide evidence that reduced activity of these new candidate X-linked driver genes could perturb normal T-cell development, confer a treatment resistance and point to a potential contribution to the observed gender bias in T-ALL among males [4].

## DISCUSSION

In this study we performed comprehensive genomic characterization of 30 pediatric T-cell ALL patients, including 7 early immature T-ALLs (of which 2 were ETP-ALLs), 13 mature T-ALLs, and 10 patients for whom the maturation status of T-ALL cells could not be determined. We identified mutations (novel and known) and analyzed expression profiles of common driver genes, further highlighting the heterogeneity and distinct characteristics of T-ALL. We identified recurrent mutations in novel childhood T-ALL genes and functionally characterized 3 new candidate driver genes (*U2AF1*, *MED12* and *USP9X*). Overall, mutation rates in this childhood T-ALL cohort were similar to those previously reported [14, 33, 35–39]. For example, 15 (50%) and 16 (53%) patients harboured *NOTCH1* mutations and *CDKN2A/2B* deletions, respectively. Immature T-ALL patients harboured significantly less *CDKN2A* deletions and experienced more relapse events compared to mature T-ALL cases, as previously published [16]. Our data also suggested a correlation between *CDKN2A* deletions and positive outcome with only one CDKN2A-deleted patient suffering a relapse event against 15 who did not, suggesting potential clinical utility of *CDKN2A* for risk-based stratification of T-ALL patients.

Another recurrently mutated T-ALL gene in our cohort was *PHF6*. Seven patients harbored *PHF6* mutations with a higher mutational rate compared to those previously reported for pediatric T-ALL (26.7% vs. 16–17.1%) [40, 33]. Interestingly, while previous analysis of the gender distribution of X-linked *PHF6* mutations showed higher prevalence among males (32.0% vs. 2.5% of females) [40], we found here a slightly higher proportion of *PHF6* mutated females (30.0% vs. 25% of males). The E3-ubiquitin ligase FBXW7 had a mutation frequency of 13.3% (*n* = 4) in our childhood T-ALL cohort, similar to previous reports (8.6–19.1%) [33, 41, 42, 35–39]. We also observed recurrent mutations in the *JAK3* oncogene (13.3% vs. 5–15.3%), which is the most frequently targeted gene of the JAK-STAT pathway in T-ALL [5, 33]. Additional known T-ALL driver genes also found to be mutated in our cohort include *KDM6A/UTX* (3.4% vs. 4.5–14%) [33, 28], *JAK1* (3.4% vs. 4.5%) [33], *PTPN2* (3.4% vs. 3.6%) [33] and *LEF1* (6.9% vs. 7.2–17.0%) [33, 43]. T-ALL genes that were mutated less frequently in our cohort, compared to previous studies, include *WT1* (6.7% vs. 13.2–16.2%) [44, 33], *PTEN* (3.4% vs. 10–11.7%) [45, 46, 5, 33] and *DNM2* (6.9% vs. 10.8– 18.0%) [5, 33]. And despite previous reports of childhood T-ALL [5, 8, 47] we found no mutation in the polycomb repressive complex 2 (PRC2), *CNOT3*, *RPL10* and *IL7R* in our cohort.

T-ALL is characteristically more common among males. Recent studies have characterized T-ALL drivers in non-autosomal regions of chromosome X such as *PHF6*, *RPL10* and *KDM6A/UTX* [40, 8, 28]. However, *PHF6* and *RPL10* are subject to X inactivation in females [40, 8], and *KDM6A/UTX* has a relatively low frequency (4.5 to 14%) in T-ALL [33, 28], therefore these genes alone cannot explain the observed skewed male:female ratio [48]. We identified additional novel X-linked candidate driver mutations in *USP9X* and *MED12* that were shown to co-occur with *PHF6* mutations in our childhood T-ALL cohort. Both genes have previously been implicated in diverse cancer types but have never been associated with T-ALL. USP9X belongs to the Ub-specific protease family and targets multiple proteins including SMURF1 [49], MCL1 [50] and Smad4 [51] and has been shown to escape X-inactivation [52]. USP9X has been demonstrated to positively regulate T-cell receptor signaling and to be required for T-cell function [53]. The oncogenic driving potential of *USP9X* was confirmed in several tumor types: loss of function of the gene in chronic myelogenous leukemia [54], hepatocellular [55] or colorectal carcinoma [56], bladder cancer [57] and B-cell ALL [58] leads to increased sensitivity to chemotherapy and to apoptosis. On the other hand, tumor suppressor functions have also been described in pancreatic adenocarcinoma [59, 60] and gingivo-buccal oral squamous cell carcinoma [61], highlighting the context-dependent role of USP9X in oncogenesis. The p.Q117* truncating mutation identified here in a single male patient (194) suggests a tumor suppressor role for *USP9X* in T-ALL, and the strong decrease in apoptosis observed following knockdown of the gene and chemotherapy drug treatment further corroborates the anti-oncogenic role of *USP9X* in childhood T-ALL and suggests a possible involvement on treatment resistance. As for *MED12*, it was altered in 10% of our T-ALL patients and was as frequently mutated as the known T-ALL gene *NRAS*. The loss-of-function mutations in *MED12* (p.V167fs and splice site g.chrX:70339329T>C) were both shown to be expressed, supporting a functional role of these mutations in T-ALL. Somatic mutations in *MED12* exon 2 splice sites have previously been identified in breast cancer and were shown to cause intron retention [62]. MED12, along with MED13, Cyclin C, and CDK8 or CDK19, is a member of the kinase module of Mediator, a multisubunit complex required for regulation of RNA polymerase II-dependent transcription [63]. In line with our observations, *MED12* was recently characterized as a tumor suppressor in uterine leiomyomas, prostate cancer, chronic lymphocytic leukemia and breast fibroadenoma [64–68]. Mutations in exon 2 showed a particularly high rate of somatic alterations (70% of uterine leiomyomas) [64] and have been shown to disrupt the direct interaction of MED12 with the cyclin C-CDK8 leading to reduced Mediator activity [66]. Here, *MED12* appears to escape X-inactivation. In addition we showed that loss of *MED12* in human T-cells leads to decreased apoptosis levels provoked by chemotherapy drugs, further corroborating its role as tumor suppressor in childhood T-ALL and highlighting its putative relapse driving potential in leukemogenesis. This is in line with recent results showing that *MED12* repression induces resistance to multiple cancer drugs through TGF-βR signaling regulation [69].

*USP9X* and *MED12* mutations were detected in the predominant clone at diagnosis and the two patients for whom we had relapse sequencing data available showed the presence of mutations in the major clone at relapse. These results indicate an early clonal selection supporting a possible functional role in early phases of T-ALL development and a possible implication in relapse. While further investigations are required to fully decipher the mechanisms underlying the observed *USP9X* and *MED12* induced anti-apoptotic effects, the identification of two novel X-linked tumor suppressor genes in pediatric T-ALL and their co-occurrence with known X-linked driver gene mutations (*PHF6*) suggests cooperating effects of X-linked mutations in T-ALL onset. The presence of several T-ALL tumor suppressor genes on the X chromosome, most of which escape X-inactivation, substantially increases the odds for males to develop the disease and could therefore explain the higher incidence of T-ALL among male children, provided that mutated forms of these proteins do not create dominant effects. However the underlying disease mechanisms associated with these alleles remains to be determined.

We also identified a novel recurrent T-ALL mutation (p.R35L) in *U2AF1* in 3 patients, including 2 immature cases. And through screening of additional adult relapsed T-ALL patients we confirmed recurrence of this novel mutation even in adults. *U2AF1* is a common mutational target in several cancer types, such as MDS where 11% of cases are mutated [70, 71]. The importance of the spliceosome machinery in leukemogenesis has been demonstrated [70, 72] and mutations in *U2AF1* were recently shown to predict poor prognosis in patients with *de novo* acute myeloid leukemia [73]. The identified p.R35L mutation was previously reported in 2 cases of myeloid neoplasms [74, 29], but to the best of our knowledge, *U2AF1* has never been associated with T-ALL prior to this study. U2AF1 has a U2AF homology motif allowing the heterodimerization with U2AF2 [75], an arginine-serine (RS) domain required for RNA high-affinity binding [76], and two ZnF domains. The p.R35L mutation identified here, as well as recurrent MDS mutations at residues S34 and Q157, fall within the ZnF domains. Although the role of these domains remains elusive, our functional studies support a function in the splicing mechanism with the p.R35L mutation leading to alternative splice site usage in known target genes such as the transcriptional corepressor BCOR, associated with poor prognosis in MDS when altered [77], or the H3K4 methyltransferase KMT2D/MLL2 that have been show to play a role in hematopoiesis [78]. KMT2C/MLL3 and KMT2D/MLL2 methyltransferases along with the histone demethylase KDM6A/UTX are members of the activating MLL2-KDM6A/UTX complex [79], their deregulation due to cooperating mutations or aberrant splicing events, could provide an alternative mechanism to recurrent alterations of PRC2 members EZH2, SUZ12 or EED in T-ALL. Mutations in *U2AF1* typically occur early in the founding clone and are present in the major clone at diagnosis [80–82], however p.R35L was subclonal in our 3 patients. This *U2AF1* mutation would appear therefore to be a late, secondary event in the developmental history of these T-ALL cases. The fact that this mutation is subclonal could explain its absence in other T-ALL studies, particularly in the event of low tumor purity or extensive intra-tumor heterogeneity. It should be noted that in ETP-ALL patient 791, p.R35L was present in a very minor subclone (VAF = 0.09) and was lost at relapse, which in this case, could render its functional role questinable. However, in addition to U2AF1 p.R35L, patient 791 also harbored a frameshift insertion p.Y816fs in the methyltransferase KMT2C/MLL3, that was carried by another subclone at diagnosis (VAF = 0.28) and presented a positive shift of frequency at relapse (VAF = 0.39). KMT2C/MLL3 and KMT2D/MLL2 methyltransferases, along with the histone demethylase KDM6A/UTX, are members of the activating MLL2-KDM6A/UTX complex involved in promoting chromatin remodeling [79]; cooperating mutations and aberrant splicing events leading to altered histone modification could contribute to disease pathogenesis.

Additional epigenetic regulators that were mutated in this childhood T-ALL cohort include: EHMT1, WHSC1, KTM2C/MLL3, CTCF, CREBBP and KDM6A/UTX. *EHMT1* codes for a H3K9 methyltransferase and is a member of the E2F6 repressor complex. To the best of our knowledge this gene has never been associated with T-ALL, although previous reports demonstrated overexpression of *EHMT1* associated with poor prognosis in esophageal cancer and treatment resistance in chronic myeloid leukemia [83, 84]. We did not identify loss-of-function mutations in known T-ALL genes *EZH2*, *SUZ12* or *EED*, but we identified activating somatic events in the H3K27 methyltransferase WHSC1 (MMSET, NSD2). *WHSC1* is a well-known and recurrent target for mutation in pediatric B-ALL as well as adult T-ALL [85, 86], however somatic disruption of *WHSC1* in pediatric T-ALL is less frequent [86]. *WHSC1* was mutated at higher frequency in our cohort, compared to previous reports in adult T-ALL (10.3% vs 4.9%) [86]. The gain of copy identified in the ETP-ALL case 791 as well as the recurrent p.E1099K mutation found in the mature case 340 lead to enhanced activation of WHSC1 which correlates with increased H3K36 and decreased H3K27 methylation and an open chromatin state across the genome. Activating *WHSC1* mutations mimic the described PCR2 loss-of-function mutations in ALL and could alter normal lymphoid differentiation and cell survival and support an oncogenic role for *WHSC1* in childhood T-ALL [87]. However the p.S231* loss of function mutation identified in the early immature patient 432 is difficult to interpret. Haploinsufficiency of *WHSC1* accounts for the core phenotypes of Wolf-Hirschhorn syndrome including facial appearance, mental retardation, growth delay and seizures [88]. This stop codon mutation could simply be a passenger event in 432 or provide a specific advantage given this patient's genetic background. The KMT2C/MLL3 frameshift mutation (p.Y816fs) in patient 791 had never been identified in hematological malignancies before. *KMT2C/MLL3* haploinsufficiency was shown to impair differentiation of hematopoietic stem and progenitor cells and to provoke resistance to conventional chemotherapy [89]. This resistance might explain the emergence of subclonal *KMT2C/MLL3* p.Y816fs positive cells in patient 791 at relapse. Finally, as recently reported in T-ALL [33], we identified the loss of a complete copy of the transcriptional repressor CTCF in one ETP-ALL patient, as well as the histone and non-histone acetyltransferase CREBBP in one mature case (636). *CTCF* was recently demonstrated to be a tumor suppressor [90] and its haploinsufficiency to lead to an increased variability in CpG methylation genome-wide. Tumors with hemizygous loss of *CTCF* showed increased aggressiveness, as observed here for ETP-ALL patient 791. The tumor suppressor gene *CREBBP* is a frequent mutational target in hematological malignancies and mutations in this gene are associated with increased risk of relapse in ALL [91], though patient 636 did not suffer relapse. Overall, these results support an important role for chromatin modification in T-ALL [92, 93, 5].

In conclusion, through integrated whole-exome, transcriptome, as well as targeted re-sequencing and genotyping investigation of 30 childhood T-ALL patients, we showed that each patient carried a unique combination of known and novel somatic alterations, including SNVs, indels, CNVs, and chromosomal rearrangements. We observed a number of uncommon and novel mutations in the early immature cases of our cohort, particularly in the two ETP-ALL patients who harbored 80% of newly-identified candidate driver mutations. We characterized a recurrent mutation in the spliceosome member U2AF1 and demonstrated its impact on alternative splicing of cancer-relevant genes, further suggesting the importance of aberrant splicing in leukemogenesis. We also identified *MED12* and *USP9X* as putative new X-linked drivers and provided evidence of the functional impact of their loss in T-cells supporting a potential role for these genes in the male-biased sex ratio observed in T-ALL. These results further highlight the underlying complexity of the genomic landscape of T-ALL, and the pressing need of larger integrative studies in well-defined cohorts to increase understanding of the biological mechanisms that contribute to T-ALL and its various subtypes.

## MATERIALS AND METHODS

### Study subjects

All study subjects were French-Canadians of European descent. Incident cases were diagnosed in the Division of Hematology-Oncology at the Sainte-Justine Hospital (Montreal, Canada) as part of the Quebec childhood ALL cohort (QcALL) [94]. This childhood T-ALL cohort (*n* = 30) consisted of 20 males and 10 females, with a mean age at diagnosis of 11.8 years. All were classified as high-risk patients and treated accordingly under FRALLE and DFCI protocols depending on year of diagnosis (Table 1). Eight patients experienced relapse after a median time of 21 months post-induction, of which five patients did not survive post-relapse, and one case (759) was refractory to induction chemotherapy and died of cerebral hemorrhage at 6 months after diagnosis.

### Whole-exome sequencing and variant identification

Whole exome sequencing (WES) was performed on 24 matched normal-tumor T-ALL patients (Figure 1). DNA was extracted from bone marrow samples (at diagnosis) and peripheral blood samples (after remission) (Table 1) using standard protocols [95]. Whole exomes were captured in solution with Agilent's SureSelect Human All Exon (38 Mb or 50 Mb), Nextera Rapid Capture Exome Enrichment kit (case 791) or SureSelectXT Clinical Research Exome (case 879) kits according to the manufacturer's protocol and sequenced on the Life Technologies SOLiD 4/5500 System (paired-end: 50 × 35 bp, mean coverage on targeted region = 35X) and for cases 791 and 879 (paired-end: 100 × 100 bp, mean coverage on targeted region = 120X). Reads obtained from SOLiD 4/5500 and HiSeq 2500 systems were aligned to the Hg19 reference genome using LifeScope Genomic Analysis Software and Bowtie2 (version 2.2.3) [96] respectively. PCR duplicates were removed using Picard [97]. Genotype quality score recalibration was performed using the Genome Analysis ToolKit (GATK) [98]. Sequencing metrics were obtained using the DepthOfCoverage option in GATK. After filtering out low quality reads, pileup files were created using SAMtools [99]. Somatic single nucleotide variants (SNVs) and small indels were called from pileup files using SNooPer, a highly versatile machine learning approach that uses Random Forest classification models and integrates matched normal-tumor data to accurately call somatic variants in low-depth sequencing data (Spinella *et al*. in revision, software available upon request).

### Targeted sequencing

Ultra-deep targeted re-sequencing was performed on all candidate somatic driver mutations using the Illumina TruSeq Custom Amplicon assay as per the manufacturer's instructions (Figure 1). Illumina DesignStudio was used to design custom oligos targeting the select mutated regions (available upon request). Genomic DNA from bone marrow at diagnosis and from blood at remission was used to validate somatic hits identified from WES (24 cases) or to screen identified variant positions in 3 additional cases with insufficient DNA for WES. PCR purification was performed with Ampure Beads for 150bp amplicon size selection. Double stranded amplicons were pooled, quantified by qPCR and sequenced on the Illumina HiSeq2500 device (paired-end: 2 × 100 bp) to reach a mean coverage of 2,500X. Reads were aligned to the Hg19 reference genome using Bowtie2 (version 2.2.3) [96]. Cleaned BAM files were used to create pileup files using SAMtools [99]. A modified version of SNooPer was used to screen mutations at the targeted positions directly in the pileup file, and to compare normal and tumoral information to confirm the somatic origin of the validated mutations (details available upon request).

Because *NOTCH1* mutations were difficult to identify in the exome sequencing data due to insufficient local coverage, Sanger sequencing was performed targeting recurrently mutated regions of *NOTCH1* in T-ALL [13]. These included the N terminal region of the heterodimerization (HD) domain on exon 26; the C terminal region of the HD domain on exon 27; the proline, glutamic acid, serine, threonine-rich (PEST) domain; and the C terminal region of the transcriptional activation domain (TAD) on exon 34. Primers used are listed in Supplementary Table S5. Chromatograms were analysed using the Sequencher software (Gene Codes) (Supplementary Figure S7).

An additional cohort consisting of 8 adult relapsed T-ALL patients from the Princess Margaret Cancer Centre, University Health Network (Toronto, Canada) (Supplementary Table S4) was used for further screening of newly identified somatic driver candidates in *USP9X*, *MED12* and *U2AF1*. Ultra-deep targeted sequencing was performed on the 5 somatic mutations identified in these 3 genes on Illumina MiSeq system (McGill University and Génome Québec Innovation Centre); primers used are available upon request. The analysis of sequencing data was performed as described above.

### RNA-sequencing and variant identification

RNA-sequencing (RNA-seq) was performed on 11 T-ALL patients (Figure 1) with suitable RNA quantity and quality. Total RNA was extracted from bone marrow samples at diagnosis for patients 432, 437, 547, 693, 716, 743, 744, 748, 791 and 849 using the mirVana Isolation kit (Ambion) according to the manufacturer's protocol. The Allprep DNA/RNA Mini kit (Qiagen) was used for relapse samples in patients 791 and 879. For patient 744, mature RNA was also purified using the Ambion's MicroPoly(A)Purist kit (Small Scale mRNA Purification Kit P/N AM1919). Following a DNAse I treatment, total or mature RNA samples were quantified by NanoDrop ND1000 (Thermo-Fisher Scientific) and RNA quality was assessed using the Agilent 2100 Bioanalyzer (Agilent). Ribosomal ribonucleic acid (rRNA) were depleted using the Invitrogen RiboMinus Eukaryote kit (Life Technologies). cDNA libraries were prepared using the SOLiD Total RNA-seq kit (diagnosis samples) and the Illumina TruSeq Stranded Total RNA kit (relapse samples) based on manufacturer's protocol and sequenced on the Life Technologies SOLiD 4/5500 System (paired-end: 50 × 35 bp) or the Illumina HiSeq 2500 System (paired-end: 100 × 100 bp). Reads obtained from SOLiD 4/5500 and HiSeq 2500 systems were aligned to the Hg19 reference genome using LifeScope Genomic Analysis Software (Whole Transcriptome Analysis pipeline, default parameters) and STAR aligner (version 2.5) [100] respectively. Remaining ribosomal sequences were filtered out. Recalibration of the genotype quality scores was performed using the Genome Analysis ToolKit (GATK) [98]. Cleaned BAM files were used to create pileup files using SAMtools [99]. SNVs and small indels identified from WES were screened in RNA-seq pileup files using a modified version of SNooPer. Reads Per Kilobase per Million mapped reads (RPKM) were calculated for 22,292 genes using the R bioconductor package edgeR [101]. Heatmaps were constructed using the heatmap.2 library of the gplots R package using RPKM values of genes of interest. Breakdancer (version 1.1.2) [102] with a minimum mapping quality (q) set to 30 was used to confirm rearrangements identified by cytogenetics or to call new events from RNA-seq data.

### Whole genome high-density SNV genotyping and copy number variant identification

Whole genome genotyping (WGG) data were available for 23 T-ALL patients (Figure 1). Normal and tumor samples from these patients were genotyped using Illumina's HumanOmni 2.5-Quad or HumanOmni2.5-Octo SNP bead arrays (McGill University and Genome Quebec Innovation Centre, Montreal, Quebec). Extracted genomic DNA was processed according to the Illumina Infinium HD Assay Ultra protocol. BeadChips were imaged on Illumina's iScan System with iScan Control Software (v3.2.45). The Genotyping Module (Version 1.9.4) of the Illumina GenomeStudio software (V2011.1) was used for raw data normalization, genotype clustering and calling, with default. ASCAT version 2.2 [103] was used to evaluate the sample purity, to evaluate tumor ploidy and to identify tumor-specific copy number variants (CNVs) or copy-neutral loss of heterozygosity (LOH).

*CDKN2A* gene allelic status was further evaluated in all T-ALL patients at diagnosis using PCR. Each 25 μl reaction contained 50 ng of template DNA, 1X KOD Buffer, 1.5 mM MgSO4, 200 μM dNTPs, 0.3 μM of each primer (listed in Supplementary Table S5) and 0.5U of KOD Hot Start DNA Polymerase (Millipore). Cycling parameters used were: 95°C 2 min; 40 cycles (95°C 20 sec, 58°C 10 sec, 70°C 10 sec). Amplified fragments of 368 bp were visualized using standard gel electrophoresis. Electrophoresis gels are available upon request.

### Variant annotation and prioritization of cancer driver gene mutations

ANNOVAR (version 2015Jun17) [104] and Oncotator (version 1.8) [105] were used to annotate somatic splice site variants, non-synonymous SNVs and frameshift small indels. Variants were queried against publically available datasets such as 1000 Genomes [106], NHLBI GO Exome Sequencing Project (ESP) [107] and Exome Aggregation Consortium (ExAC) [108] to filter out common polymorphisms (minor allele frequency > 0.01). The Catalogue of Somatic Mutations in Cancer (COSMIC, version 72) [109] was used to evaluate prior implications in cancer. We classified each mutated gene as either tumor suppressor genes (TSGs) or oncogenes based on Vogelstein's 20/20 rule [110] on COSMIC v72 data (Supplementary Table S3). The predicted functional impact of non-synonymous variants and small indels was assessed using Sift (version 1.03) [111], Polyphen2 (version 2.2.2) [112], MutationTaster 2 [113] and the Cancer-specific High-throughput Annotation of Somatic Mutations tool (CHASM version 3.0) [114] (Supplementary Table S3). Variants presenting a score ≤ 0.05 were considered as damaging by Sift (D). Mutations with a Polyphen2 score between 0.447 and 0.909 were predicted as possibly damaging (P) while a score > 0.909 was considered as damaging. Mutations with a MutationTaster score > 0.9 were also considered as damaging. Finally, for CHASM classification we used the Blood-Lymphocyte training set and a Benjamini and Hochberg's adjusted false discovery rate (FDR) ≤ 0.20, to prioritize mutations based on their predicted driver potential. To identify candidate driver mutations we filtered events and kept only somatic alterations that were: i) missense mutations predicted to be driver by CHASM [114]; or to be damaging by at least two of the other three prediction algorithms; or identified as recurrent in COSMIC v72 [109]; ii) splice site and nonsense mutations that were predicted to be damaging by MutationTaster and Sift, respectively; or located in a tumor suppressor gene; or identified as recurrent in COSMIC; iii) frameshift indels and deletions located in a TSG and gain of copies located in an oncogene; iv) genes with more than one mutation (SNV, indel or CNV) in our cohort but previously never associated with pediatric T-ALL were considered as new candidate driver genes. Based on these filtering criteria, we identified somatic gene mutations with putative functional effects in driving T-ALL development, some of which we further investigated (*USP9X, MED12* and *U2AF1*).

### Cell lines

Human acute T-cell leukemia-derived Jurkat Tet-On cells (630915, Clontech) and REH human leukemia cells (#CRL-8286, A.T.C.C.) were grown in RPMI-1640 medium (Wisent) supplemented with 10% fetal bovine serum, 100 IU/ml penicillin and 100 μg/ml streptomycin (Wisent). The highly transfectable HEK293T cells were grown in Dulbecco's Modified Eagle's Medium supplemented with 10% fetal bovine serum (Wisent). Cells were routinely maintained at 37°C in a humidified atmosphere composed of 95% air and 5% CO2 and provided with fresh medium every 2 to 3 days.

### RT-PCR validation of alternative splicing in MED12

Total RNA was extracted (as above) from the patient's bone marrow at diagnosis, as well as from REH (human B leukemia) cells, mature T cells (CD3+/CD19-) isolated from cord blood samples, and from two g.chrX:70339329TT wild type (WT) patients (727 and 791), used as controls. RNA was reverse transcribed into cDNA using the Ovation^®^ qPCR System (NuGEN Technologies). PCR were performed using KOD Polymerase as described above. Amplified fragments were analyzed on the Agilent 2100 Bioanalyzer Instrument and by Sanger sequencing (McGill University and Genome Quebec Innovation Centre).

### ShRNA-mediated gene knockdown

Lentivirus-mediated gene-specific small hairpin RNAs (shRNAs) were used to knockdown expression of 2 candidate driver genes: *USP9X*, and *MED12*, in Jurkat (human T leukemia) cells. The complete list of MISSION^®^ shRNAs (Sigma-Aldrich) used to silence target gene expression are listed in Supplementary Table S6 (at least 3 for each gene). shRNA target sequences were subcloned into either the lentiviral vector pLKO.1-puro (TRC1 version) or pLKO.5-puro (TRC2 version). Briefly, using Polyethylenimine (PEI; Polysciences), plasmids and packaging vectors (6 μg of pRSV-Rev, 7.8 μg of plasmid pMD2.VSVG, 15 μg of pMDL, and 9 μg of shRNA in pLKO-puro plasmid) were co-transfected into HEK293T cells to generate respective lentivirus. Supernatants containing lentiviruses were harvested 48 h post-transfection. 1×10E6 Jurkat cells were infected with 1 ml of supernatant in the presence of 5 μg/mL of polybrene (Sigma-Aldrich). 72 hours post-infection, cells were screened with 2 μg/μl puromycin (Sigma-Aldrich) for two weeks to select for shRNA-knockdown cells. Three biologically independent replicates were carried out for each target gene. The expression of target genes was measured by quantitative PCR (qPCR).

Total RNA was extracted from infected cells using RNeasy mini kit (Qiagen). 500 ng of total RNA were reverse transcribed using the M-MLV reverse transcriptase (Thermo Fisher Scientific) and qPCR amplifications (triplicates) were performed on the ABI PRISM 7000 Sequence Detection System (Thermo Fisher Scientific) in a total volume of 25 μl as follows: 5 μl of cDNA (diluted 1:5), 0.2 μM of each primers (listed in Supplementary Table S5), and 1X SYBR Green PCR Master Mix (Thermo Fisher Scientific). The cycling parameters were: 95°C 10 min; 40 cycles [95°C 15 sec, 62°C 1 min], followed by a denaturation curve at 60°C. GAPDH was used as reference gene. Expression values were calculated by the 2^−(ΔΔCt)^ formula previously described [115].

### Apoptosis assay

Apoptosis was measured using the Alexa Fluor^®^ 488 Annexin V/Dead Cell Apoptosis Kit (Thermo Fisher Scientific) according to the manufacturer's instructions. Briefly, cells were seeded at 5×10E5 cells/mL in their culture medium and treated with 2 μM of Camptothecin for 17 hours or 500 nM of Doxorubicine for 18 hours or 50 nM of Vincristine for 24 hours, to promote DNA damage-induced apoptosis. Following incubation with Annexin V Alexa Fluor^®^ and propidium iodide (PI) at room temperature for 30 min, stained cells were immediately analyzed by flow cytometry. The percentage of apoptotic cells was measured on the FACS Fortessa using the BD FACSDiva software (BD Biosciences) according to manufacturer's guidelines. At least three independent experiments were carried out for each biological replicate.

### U2AF1 p.R35L splicing assay

The pOTB7 plasmid containing *U2AF1* cDNA was purchased from Harvard PlasmID Repository (clone # HsCD00321863) and subcloned into the Gateway compatible vector pDONR221. The R35L mutation was introduced into the cDNA sequence of *U2AF1* using the QuickChange II XL Site Directed Mutagenesis kit (Agilent) with primers listed in Supplementary Table S5. Sanger sequencing (McGill University and Genome Quebec Innovation Centre) was performed to confirm the presence of the mutation. WT or R35L *U2AF1* coding sequences were then subcloned into the Gateway lentiviral vector pLenti CMV Puro DEST (w118–1) using LR clonase Enzyme mix (Thermo Fisher Scientific). Lentiviruses were generated and Jurkat cells infected (two biologically independent replicates) as described above. Total RNA was extracted from infected cells using RNeasy Mini Kit and treated with RNase-Free DNase Set (Qiagen), and cDNAs were generated using M-MLV reverse transcriptase (Thermo Fisher Scientific). Overexpression of WT or R35L *U2AF1* in Jurkat cells was measured by qPCR amplification as described above. To validate alternative splice site utilization at the *BCOR* and *KMT2D/MLL2* target genes, as described elsewhere [32], RT-PCR was performed on cDNAs in duplicates using KOD Hot Start Polymerase as described previously with primers listed in Supplementary Table S5. PCR products were electrophoresed in agarose gel stained with SYBR Safe (Thermo Fisher Scientific), and quantified by densitometry using Image J software (version 1.49).

### Statistical tests

Significance of observations was assessed with R using two-tailed Fisher's exact test or Mann-Whitney-*U* test when appropriate.

## SUPPLEMENTARY MATERIALS FIGURES AND TABLES














