# Supplementary material for: Genomic characterization of pediatric T-cell acute lymphoblastic leukemia reveals novel recurrent driver mutations
Source: Oncotarget. 2016 Sep 1;7(40):65485–503. doi: 10.18632/oncotarget.11796 (PMC5323170; doi:10.18632/oncotarget.11796)
Supplement: Supplementary file 1 [file oncotarget-07-65485-s001.pdf]

Genomic characterization of pediatric T-cell acute lymphoblastic leukemia reveals novel recurrent driver mutations

Supplementary Materials

SUPPLEMENTARY INFORMATION

Classification of T-ALL patients by maturation stage

We used both immunophenotyping and gene expression data when available to classify patients according to T-cell maturation status (Table 1, Supplementary Figure S1, Supplementary Table S1 and Supplementary Table S2). Seven patients (324, 432, 706, 716, 748, 791 and 879) clustered as early immature T-ALLs. Five of these tumors strongly expressed CD34 and most had no or weak expression of mature thymocyte markers CD1a, CD4 and CD8. These CD34+/CD1a−/CD4−/CD8− patients also expressed immature T-cell specific markers such as *LMO2, LYL1, BCL2, FLT3, TGFB1* and *MYB* [1]. Two of these patients (791 and 879), had low expression levels of CD5 (< 75%) and were positive for myeloid or stem-cell markers CD34, HLA-DR, CD13, CD33, CD11b and positive for the T-cell marker cytoplasmic CD3 (cCD3). They expressed *LYL1*, indicative of an early T-cell precursor ALL (ETP-ALL) phenotype [2]. Twelve patients (340, 341, 437, 544, 547, 636, 693, 727, 743, 744, 759 and 849) positive for CD1a/CD4/CD8 and expressing mature thymocyte markers such as *LCK, RAG1, PTCRA* or *STAT5A* [1] were classified as mature T-ALLs (Supplementary Figure S1 and Supplementary Table S1). Among these, patients 744 and 849 showed the strongest ectopic expression of the homeobox developmental genes *SIX6* and *NKX3*-1 [1], and patient 849 showed strong activation of the TAL1 oncogene, further supporting a more advanced (late cortical) stage of maturation. Of note, CD1a+/CD4+ patient 693 highly expressed *HOXA* genes indicative of T-lineage blockade prior to beta-selection [1], suggesting an early cortical stage of maturation for this mature T-ALL. Some patients were difficult to classify, such as patient 744 who was classified as mature T-ALL and was the only patient who showed activation of *TLX3*, typically associated with early cortical cases. However patient 744 was CD34+ and also expressed immature markers such as *FLT3* and *BMI1*.

Chromosomal rearrangements and cryptic events in T-ALL

We identified translocations using Fluorescent In Situ Hybridization (FISH), molecular cytogenetic analyses and RNA-seq data. RNA-seq data confirmed aberrant transcriptional activation of *TAL1* for patient 849 but surprisingly showed no up-regulation of this gene in patient 547 (Supplementary Figure S1A). This is possibly due to intra-tumor heterogeneity of the sample leading to reduced tumor-specific gene expression signals. We could not confirm upregulation of *LMO2* and *TLX1/HOX11* in patients 759 and 636 because of the lack of expression data. Transcriptome analysis of the two ETP-ALL cases with the t(10;11)(p12:q14) CALM-AF10 translocation revealed aberrant activation of the *HOXA* cluster [3], as well as activation of its cofactors BMI1 and MEIS1 (Supplementary Figure S1A and Supplementary Table S2). Of note, *BMI1* activation leads to *CDKN2A* inhibition and thus induces cell proliferation, which corroborates with the absence of *CDKN2A* locus deletions in these immature T-ALL patients [4]. Only one patient (744) showed cryptic activation of *TLX3*, typically associated with early cortical cases. This patient also had upregulation of *BMI1*, but no characteristic co-upregulation of *HOXA* genes was observed, nor did we identify CALM-AF10 or MLL rearrangements typically causing *BMI1* activation [3]. Patient 693 presenting the overall strongest activation of the *HOXA* gene cluster also showed upregulation of oncogenes and T-cell differentiation genes such as *NOTCH2, NOTCH3, PTCRA* and *PIM1* as expected. However no expression of *LMO2, LYL1, BCL2* or *FLT3* was observed in this patient. Of note, *TLX1/HOX11* activation, associated with early cortical T-ALLs and with a favorable prognosis [4], was not observed in any of the patients investigated here.

Clonal architecture of childhood T-ALL

We used genomic sequencing data (read counts) to study clonal architecture of these childhood T-ALL tumors. For both ETP-ALL patients, we also investigated clonal dynamics from diagnosis (pre-treatment) to relapse. Variant allele frequencies (VAFs) of 48 somatic SNVs and small indels from 66 candidate drivers were estimated from Illumina WES (Methods, mean coverage on targeted region = 120×) and/or ultra-deep targeted re-sequencing data (Methods, mean coverage = 2,500×). Variants identified from RNA-seq data and Sanger sequencing were not considered. Tumor purity was determined either from blast counts at diagnosis or from ASCAT profiles (Table 1), and was used to adjust VAFs in each tumor

(Supplementary Figure S3 and Supplementary Table S3). Frequencies were not adjusted for copy number variations, which would explain the high somatic variant frequencies at certain loci that overlapped monoallelic deletion events (e.g. *WT1* p.R370H (VAF = 0.96)). Once adjusted, 29.2% of identified somatic SNVs and small indels were considered as subclonal as they presented adjusted VAFs ≤ 0.4, and of VAFs ≤ 0.8 for X-linked mutations in males (Supplementary Figure S3A and Supplementary Table S3). Variations in common T-ALL drivers (*PHF6, FBXW7, JAK1* and *JAK3*) were mostly clonal (mean VAF = 0.48, standard dev. = 0.10). Interestingly, both variations in *JAK1* and *JAK3* identified in patient 744 were clonal (VAF = 0.59 and 0.56, respectively), suggesting an early cooperative role as opposed to previous reports of a sequential event with *JAK1* acting as first hit and *JAK3* contributing to selection and expansion of the *JAK1*+ subclone [5]. Ras pathway mutations had significantly lower frequencies compared to these common drivers with a mean VAF = 0.33 (standard dev. = 0.11) ($p = 0.006$, Mann-Whitney-*U* test). Subclonality of these mutations corroborates previous reports [6–8] that describe a secondary role for Ras mutations in T-ALL occurring later in tumor progression. SNVs and small indels identified in novel T-ALL genes had different modalities of clonal evolution (Supplementary Figure S3A and Supplementary Table S3). For *U2AF1*, p.R35L was subclonal in all three patients carrying the mutation (mean VAF = 0.24, standard dev. = 0.13), and was therefore likely recently acquired in tumor evolution. On the other hand, mutations in newly identified X chromosome genes *USP9X* and *MED12* (mean = 0.97, SD = 0.04) in patients 194, 744, 791 and 879 had VAFs that were similar to the known driver mutations in *PHF6* and *KDM6A/UTX* (mean = 0.97, SD = 0.05) ($p = 1.0000$). Given that we had quality relapse data for the two ETP-ALL patients (791 and 879), we investigated the mutational trajectories of known and novel candidate driver genes (Supplementary Figure S3B and Supplementary Figure S3C and Supplementary Table S3). The CALM-AF10 translocation carried by both patients at diagnosis was also found in the dominant clone at relapse. In addition to the dominant *PHF6* (p.G226fs, VAF = 0.51) and *MED12* (p.R1989H, VAF = 0.49) mutations, case 791 also carried subclonal mutations in *KMT2C/MLL3* (p.Y816fs, VAF = 0.28) and *JAK3* (p.M511I, VAF = 0.13). While patient 791's most subclonal mutations (*NOTCH1*, VAF = 0.10; JAK3, VAF = 0.13; *U2AF1*, VAF = 0.09) were lost at relapse, the subclonal tumor cell population carrying the *KMT2C/MLL3* mutation at diagnosis, was selected at relapse with a positive VAF shift from 0.28 to 0.39. On the other hand, X-linked mutations remained clonal with relapse VAFs of 0.46 and 0.44 for *PHF6* (p.G226fs) and *MED12* (p.R1989H) respectively. This suggested the presence of multiple subclones at diagnosis and subsequent purification at relapse with the emergence of a single founder clone. As for patient 879, almost all relapse mutations were present in the major clone at diagnosis and remained clonal at relapse, including *PHF6* (p.R225*, VAF = 0.55 to 0.49), *MED12* (p.V167fs, VAF = 0.55 to 0.49), *JAK3* (p.L857P, VAF = 0.54 to 0.53) and *FBXW7* (p.W425C, VAF = 0.55 to 0.45). Only *WT1* (p.V167fs) was counter-selected at relapse and presented a negative VAF shift from 0.49 to 0.29. Overall, subclones present in both patients at diagnosis underwent a putative selection illustrated by the loss of subclones in 791 with the emergence of a fitter dominant clone at relapse carrying *KMT2C* p.Y816fs, and loss of a *WT1* p.V167fs subclone in 879. Interestingly, no additional relapse-specific events were identified in these cases. Given that *MED12* positive cells showed clonal equilibrium, it was difficult to evaluate their relapse potential, however their maintenance as dominant population in both cases suggests potential involvement in the process.

REFERENCES

1. Soulier J, Clappier E, Cayuela JM, Regnault A, García-Peydró M, Dombret H, Baruchel A, Toribio ML, Sigaux F. HOXA genes are included in genetic and biologic networks defining human acute T-cell leukemia (T-ALL). Blood. 2005; 106:274–86.
2. Coustan-Smith E, Mullighan CG, Onciu M, Behm FG, Raimondi SC, Pei D, Cheng C, Su X, Rubnitz JE, Basso G, Biondi A, Pui CH, Downing JR, et al. Early T-cell precursor leukaemia: a subtype of very high-risk acute lymphoblastic leukaemia. Lancet Oncol. 2009; 10:147–56.
3. Caudell D, Aplan PD. The role of CALM-AF10 gene fusion in acute leukemia. Leukemia. 2008; 22:678–85.
4. Ferrando AA1, Neuberg DS, Staunton J, Loh ML, Huard C, Raimondi SC, Behm FG, Pui CH, Downing JR, Gilliland DG, Lander ES, Golub TR, Look AT. Gene expression signatures define novel oncogenic pathways in T cell acute lymphoblastic leukemia. Cancer Cell. 2002; 1:75–87.
5. Vicente C, Schwab C, Broux M, Geerdens E, Degryse S, Demeyer S, Lahortiga I, Elliott A, Chilton L, La Starza R, Mecucci C, Vandenberghe P, Goulden N, et al. Targeted sequencing identifies association between IL7R-JAK mutations and epigenetic modulators in T-cell acute lymphoblastic leukemia. Haematologica. 2015; 100:1301–10.
6. Tartaglia M, Martinelli S, Cazzaniga G, Cordeddu V, Iavarone I, Spinelli M, Palmi C, Carta C, Pession A, Aricò M, Masera G, Basso G, Sorcini M, et al. Genetic evidence for lineage-related and differentiation stage-related contribution of somatic PTPN11 mutations to leukemogenesis in childhood acute leukemia. Blood. 2004; 104:307–13.
7. Case M, Matheson E, Minto L, Hassan R, Harrison CJ, Bown N, Bailey S, Vormoor J, Hall AG, Irving JA. Mutation of genes affecting the RAS pathway is common in childhood acute lymphoblastic leukemia. Cancer Res. 2008; 68:6803–9.

8. Irving J, Matheson E, Minto L, Blair H, Case M, Halsey C, Swidenbank I, Ponthan F, Kirschner-Schwabe R, Groeneveld-Krentz S, Hof J, Allan J, Harrison C, et al. RAS pathway mutations are highly prevalent in relapsed childhood acute lymphoblastic leukaemia, are frequently relapse-drivers and confer sensitivity to MEK Inhibition. Blood. 2013; 122:823.

9. Han X, Bueso-Ramos CE. Precursor T-cell acute lymphoblastic leukemia/lymphoblastic lymphoma and acute biphenotypic leukemias. Am J Clin Pathol. 2007; 127:528–44.

10. Vogelstein B, Papadopoulos N, Velculescu VE, Zhou S, Diaz Jr. LA, Kinzler KW. Cancer Genome Landscapes. Science. 2013; 339:1546–58.

11. Shirai CL, Ley JN, White BS, Kim S, Tibbitts J, Shao J, Ndonwi M, Wadugu B, Duncavage EJ, Okeyo-Owuor T, Liu T, Griffith M, McGrath S, et al. Mutant U2AF1 Expression Alters Hematopoiesis and Pre-mRNA Splicing *In Vivo*. Cancer Cell. 2015; 27:631–43.

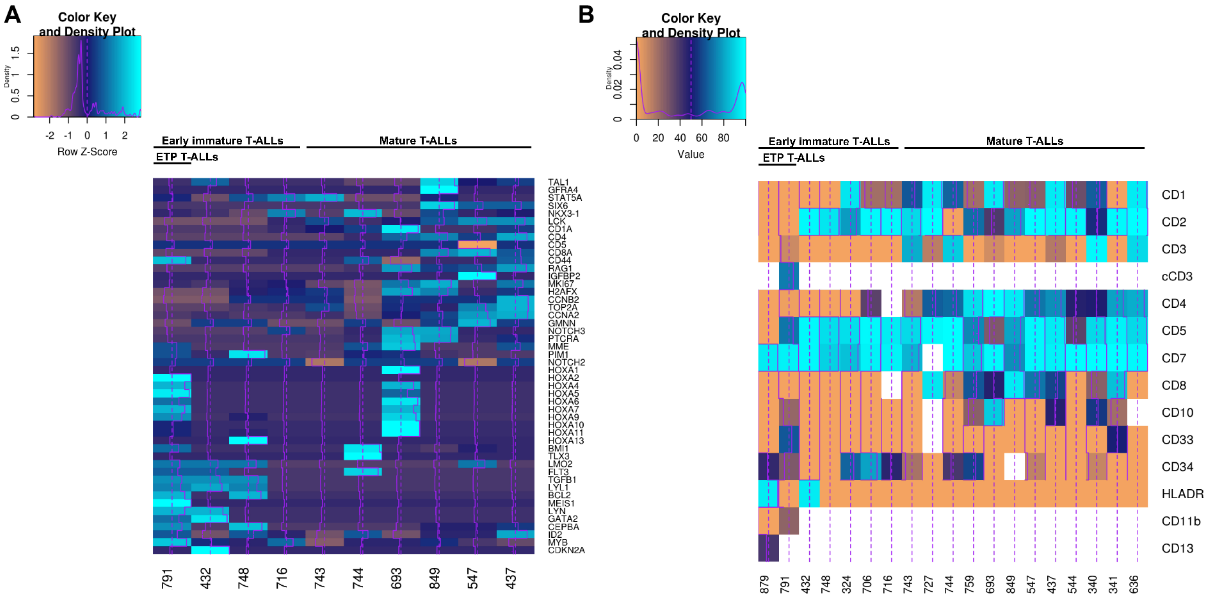

Supplementary Figure S1: Genes and antigenic determinant expression for the classification of T-ALL cases. (**A**) Informative markers were selected based on previously published classification criteria [1, 2, 9]. Z-scores were calculated based on Reads Per Kilobase per Million mapped reads (RPKM) obtained from RNA sequencing data (SOLiD 4/5500 System) using the R bioconductor package edgeR (Methods) and scaled per selected marker (red lines). Dotted lines are centered on 0. (**B**) Informative antigenic determinants. Values are percentages of positive leukemic cells for each determinant (black lines). Dotted lines are centered on 50%. White cells represent missing values. Scales for color density are indicated in the top left corner of each heatmap.

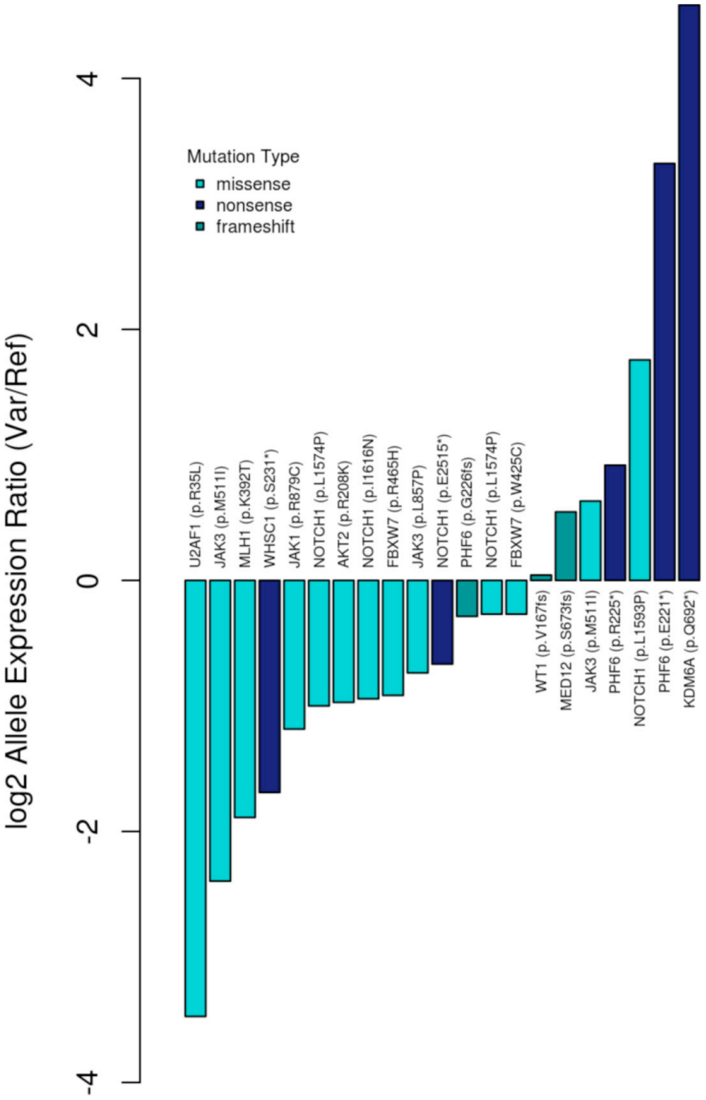

Supplementary Figure S2: The analysis of tumor transcriptomes reveal the expression of 84% of identified somatic events (SNVs and Small Indels). Log2 ratios of the number of RNA-seq reads supporting the variant and the reference allele for each expressed somatic event.

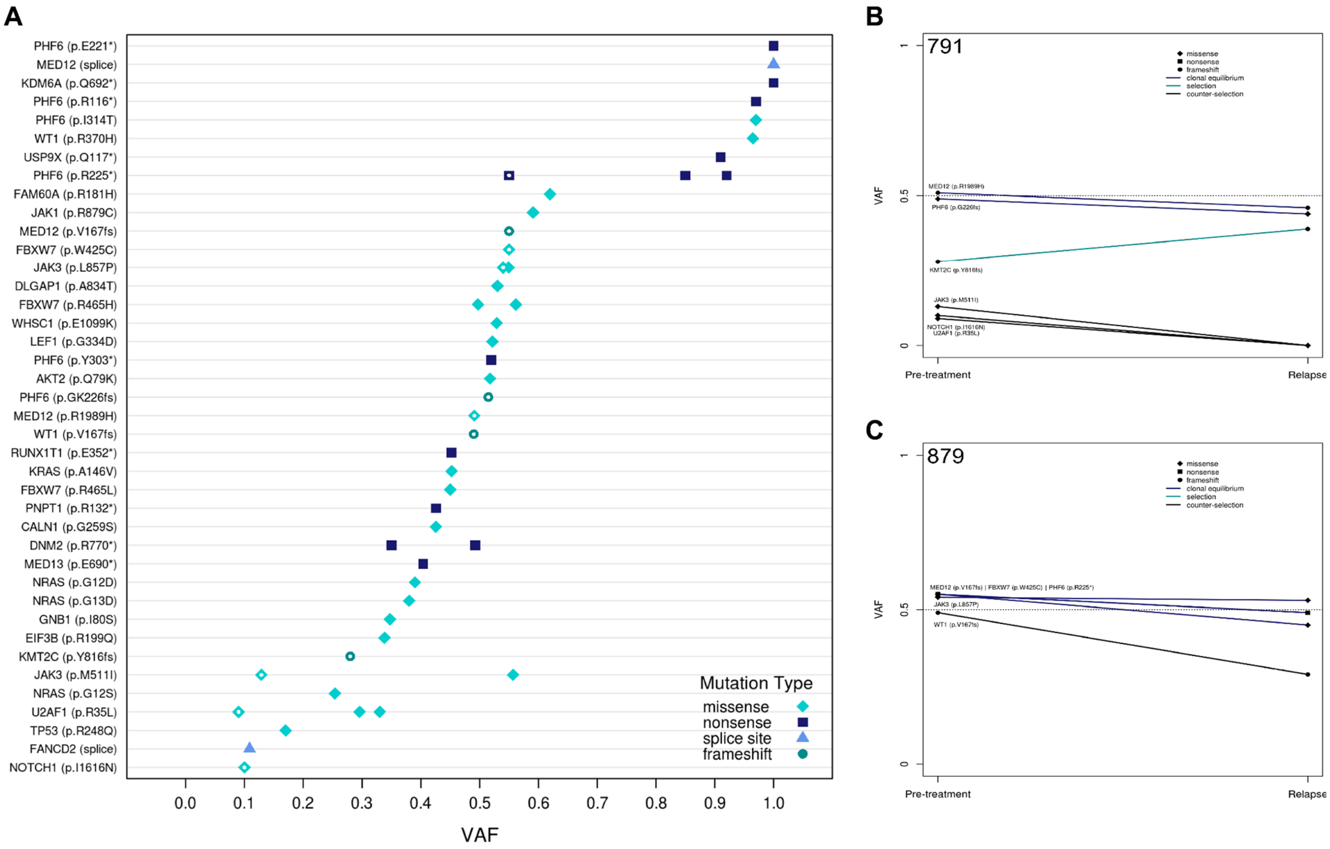

Supplementary Figure S3: Variant Allele Frequency (VAF) analysis at diagnosis and relapse reveals the clonal architecture of somatic events (SNVs and Small Indels) and their evolution under the selection pressure of therapy. (**A**) VAFs of somatic events correspond to the ratio of reads supporting the mutation over the total depth of coverage at the given position. VAFs were calculated from ultra-deep targeted re-sequencing (mean coverage of 2,500×) and adjusted according to either tumor purity (blast count) or from the analysis of genotype profiles using ASCAT. Amino acid changes are indicated in brackets beside gene names. Squares, circles or diamonds with a central white dot indicate events identified at diagnosis from patients with relapse data available (791 and 879). *: stop gain; fs: frameshift. (**B**) Clonal dynamics from diagnosis (pre-treatment) to relapse of the ETP-ALL cases 791 (upper panel) and 879 (lower panel). VAFs at relapse are calculated from WES data and adjusted according to tumor purity. Dark blue, green and black lines stand for clonal equilibrium, positive shift and negative shift respectively.

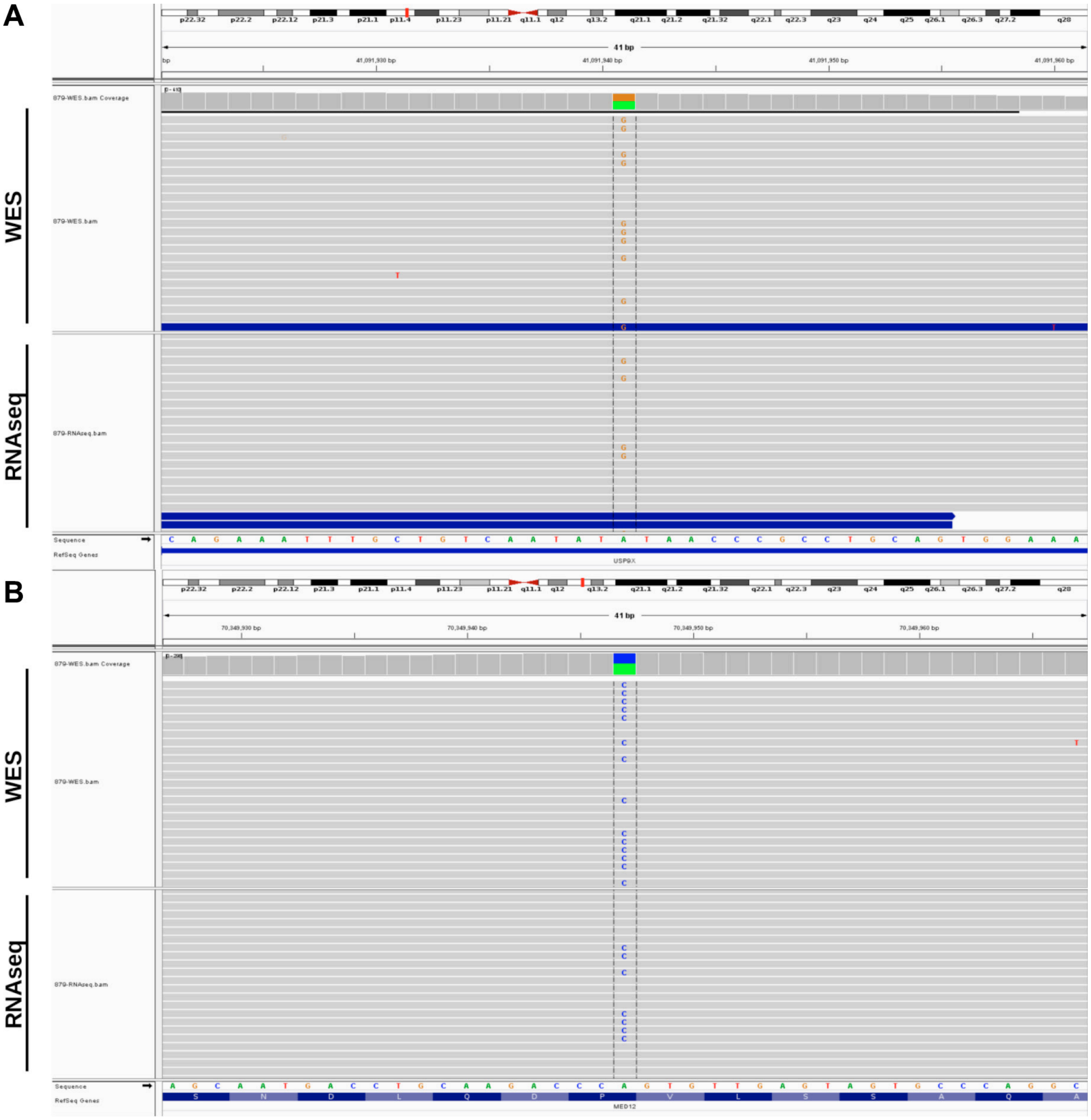

Supplementary Figure S4: *USP9X* and *MED12* escape X-inactivation in patient 879. Screenshot of the Integrative Genomics Viewer (IGV) window presenting aligned RNA-seq data obtained from patient 879. The dotted lines are centered on the informative SNPs rs10463 (**A**), located in 3′UTR of *USP9X* as control, and rs5030619 (**B**), located in exon 28 of *MED12* (bottom). Both positions are covered by reference and supporting reads.

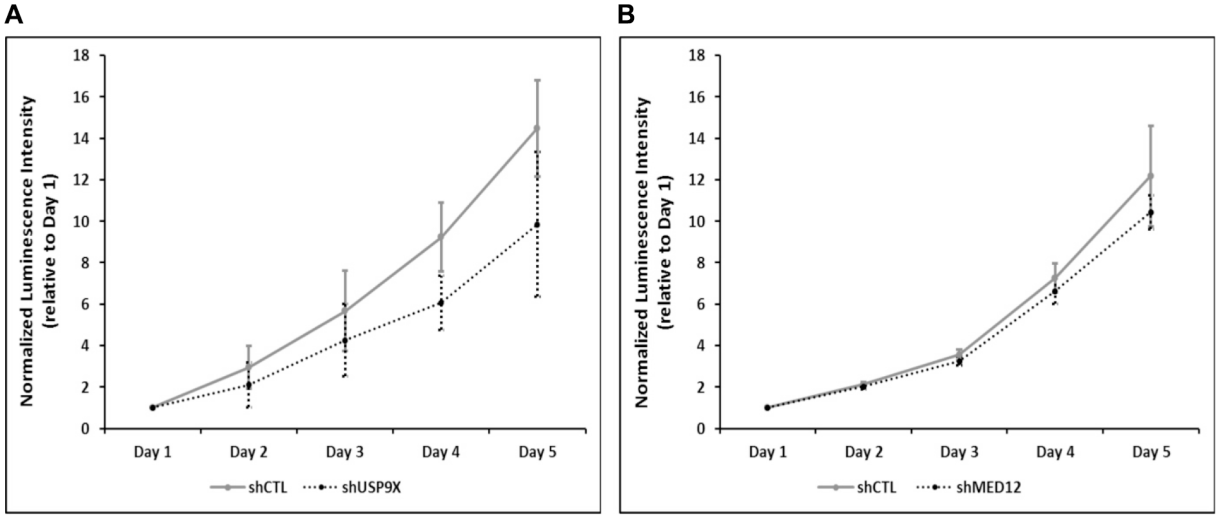

Supplementary Figure S5: The loss of function USP9X and MED12 has no effect on cell proliferation in Jurkat cells. The experiment was performed using CellTiter-Glo® Luminescent Cell Viability Assay. 1 × 10E4 cells were seeded in triplicates, harvested daily over 5 days, mixed with CellTiter-Glo solution, and resulting luminescence read on Envision plate reader. Jurkat cells transduced with shUSP9X (**A**) and shMED12 (**B**) were compared to Jurkat cells transduced with non-mammalian shRNAs (shCTL). Normalization was performed daily for each replicate by comparison to data obtained during corresponding day 1.

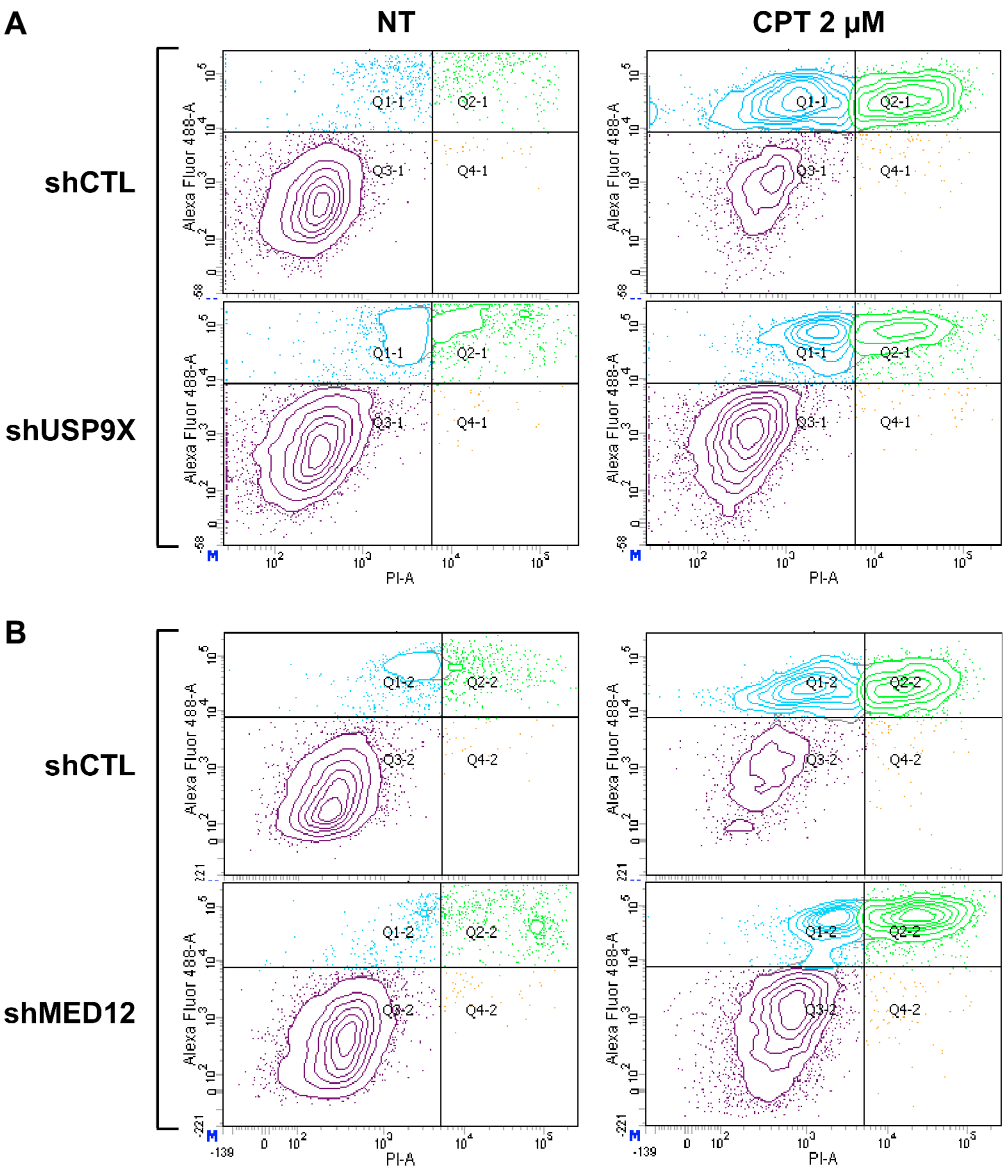

Supplementary Figure S6: The loss of function of USP9X and MED12 protects from camptothecin-induced apoptosis in leukemic T-cells. Representative flow cytometry profiles of apoptosis assays performed on Jurkat cells infected with shRNAs targeting USP9X (**A**) and MED12 (**B**), non-treated (NT) or treated with 2 µM Camptothecin (CPT) for 17 h, compared to Jurkat cells transduced with non-mammalian shRNAs (shCTL). Staining was performed using Alexa Fluor® 488-conjugated Annexin V and PI for 30 minutes. Data shown are representative of three independent shRNA infections.

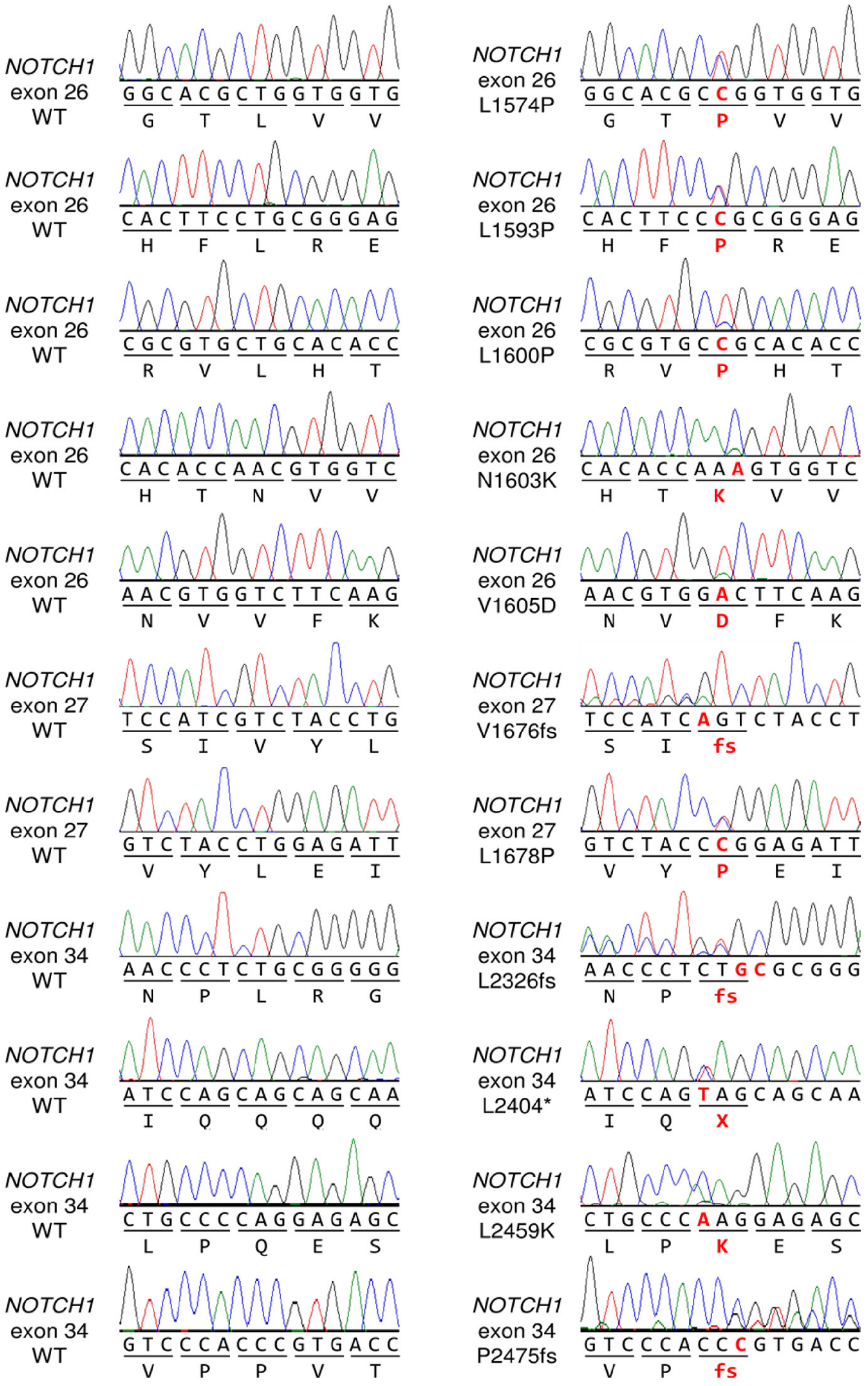

Supplementary Figure S7: Sanger sequencing for the identification of mutations in *NOTCH1*. Representative DNA sequencing chromatograms of tumoral genomic DNA samples showing somatic mutations in exons 26, 27 and 34 of *NOTCH1* (Methods).

Supplementary Table S1: List of Informative antigenic determinants. See Supplementary_Table_S1

Supplementary Table S2: Gene expression for the classification of the 2 ETP T-ALL cases. See Supplementary_Table_S2

Supplementary Table S3: Identified SNVs, Small Indels and CNVs among 30 childhood T-ALL patients. See Supplementary_Table_S3

Supplementary Table S4: Additional cohort of 8 adult relapsed T-ALL patients. See Supplementary_Table_S4

Supplementary Table S5: List of primers. See Supplementary_Table_S5

Supplementary Table S6: List of shRNAs. See Supplementary_Table_S6
